# Advances in machine learning and IoT for water quality monitoring: A comprehensive review

**DOI:** 10.1016/j.heliyon.2024.e27920

**Published:** 2024-03-13

**Authors:** Ismail Essamlali, Hasna Nhaila, Mohamed El Khaili

**Affiliations:** Electrical Engineering and Intelligent Systems Laboratory, ENSET Mohammedia, Hassan 2nd University of Casablanca, Mail Box 159, Morocco

**Keywords:** Water quality, Wireless IoT technology, Machine learning, Environmental monitoring

## Abstract

Water holds great significance as a vital resource in our everyday lives, highlighting the important to continuously monitor its quality to ensure its usability. The advent of the. The Internet of Things (IoT) has brought about a revolutionary shift by enabling real-time data collection from diverse sources, thereby facilitating efficient monitoring of water quality (WQ). By employing Machine learning (ML) techniques, this gathered data can be analyzed to make accurate predictions regarding water quality. These predictive insights play a crucial role in decision-making processes aimed at safeguarding water quality, such as identifying areas in need of immediate attention and implementing preventive measures to avert contamination. This paper aims to provide a comprehensive review of the current state of the art in water quality monitoring, with a specific focus on the employment of IoT wireless technologies and ML techniques. The study examines the utilization of a range of IoT wireless technologies, including Low-Power Wide Area Networks (LpWAN), Wi-Fi, Zigbee, Radio Frequency Identification (RFID), cellular networks, and Bluetooth, in the context of monitoring water quality. Furthermore, it explores the application of both supervised and unsupervised ML algorithms for analyzing and interpreting the collected data. In addition to discussing the current state of the art, this survey also addresses the challenges and open research questions involved in integrating IoT wireless technologies and ML for water quality monitoring (WQM).

## 1 Introduction

Water serves as a crucial natural resource, sustaining life on our planet. Ensuring the quality of water is imperative for the well-being of humans, the preservation of the environment, and fostering economic growth. However, the global community faces formidable obstacles in effectively monitoring and managing water resources. The availability of safe and clean drinking water is not only a basic human right but also a pivotal factor in promoting public health and sustainable development. While progress has been made in enhancing access to improved water sources, the prevalence of water scarcity remains worrisome, affecting a significant proportion of the global population, estimated at around four billion people, for at least one month annually [1]. The factors influencing water quality (WQ) are intricate and diverse, encompassing natural phenomena such as erosion and sedimentation, as well as human activities like agriculture, industry, and urbanization.

Furthermore, challenges are compounded by the exacerbating impacts regarding climate change and population increase. Water pollution has severe consequences, as it leads to the outbreak of waterborne illnesses like cholera, dysentery, and typhoid fever, posing significant threats to human health. Moreover, the adverse effects of poor WQ extend beyond individual well-being, impacting and impeding economic development. The United Nations' Sustainable Development Goal 6 (SDG 6), titled "Clean Water and Sanitation," is a crucial initiative designed to ensure access to safe and clean drinking water and proper sanitation facilities for all people worldwide. SDG 6 encompasses targets related to enhancing WQ, expanding access to clean water sources, improving sanitation services, and addressing issues related to water scarcity and pollution. This goal plays a vital role in promoting public health, reducing waterborne diseases, and fostering overall sustainable development [2].

**Fig. 1:**
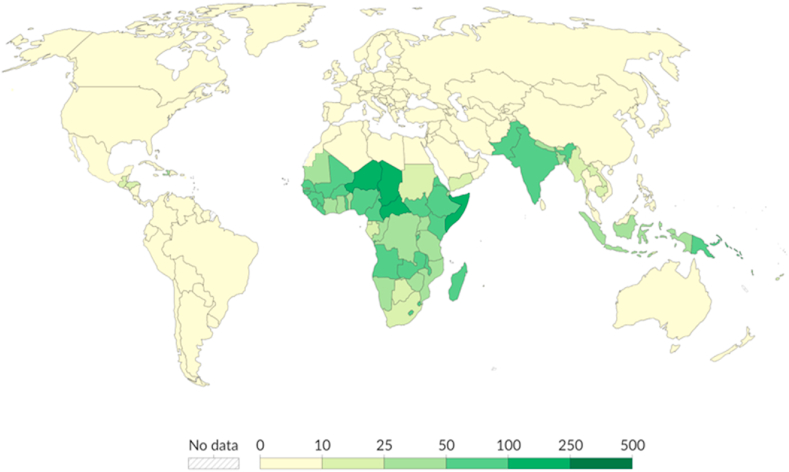
Estimated annual number of deaths related to unsafe water per 100,000 people (2019) [6].

Water is susceptible to the impacts of climate change. The research conducted by Duran-Encalada et al. [3] reveals that climate change can significantly affect both the quantity and quality of available water, in addition to other water features. Furthermore, the exacerbation of this issue is evident due to the escalating demand of the increasing population, which has led to a decline and degradation of receiving surface water [4], as well as a reduction in the chemical WQ of rivers [5]. As a consequence, a significant number of individuals, particularly in developing nations, suffer fatal consequences due to water-related issues as illustrated in Fig. 1 [6].

Water quality monitoring (WQM) is a crucial task that requires an interdisciplinary approach and involves the understanding of factors that impact WQ, the development of strategies to enhance and manage water resources, and the implementation of policies and regulations to regulate human activities that can potentially affect WQ. Moreover, engaging local communities and stakeholders, as well as promoting public awareness and education about the importance of WQ, is also vital for successful WQM.

The continuous development of IoT technology has revolutionized the WQM, making it more streamlined and affordable. The utilization of IoT devices has gained significant traction in recent times and is anticipated to persist as a growing trend. The global count of connected devices is estimated to reach 30.9 billion by 2025, signifying a noteworthy surge in the adoption of IoT technology to monitor WQ [7]. IoT devices growth has been facilitated by low-cost, high-performance hardware, and wireless communication technologies, which have made it easier and more affordable to deploy these devices. The increasing adoption of IoT technology is expected to have a significant impact on WQM, as more devices are deployed to monitor WQ parameters in real-time, leading to better protection and management of our water resources.

IoT wireless technologies, such as Wi-Fi, RFID, LpWAN, and cellular networks, have revolutionized WQM, allowing for the remote and real-time monitoring of WQ parameters. This eliminates the need for physical sampling and analysis, resulting in improved accuracy and reliability of WQM. Additionally, remote monitoring enables proactive and timely responses to any anomalies or changes in WQ, which ultimately leads to better protection of our water resources. IoT technology offers advantages over traditional WQM methods by enabling continuous remote monitoring for real-time anomaly detection, providing cost-effective and comprehensive coverage, and collecting data for analysis and informing management strategies.

The use of IoT devices for WQM alone does not guarantee improved WQ. To improve WQM, the data collected through these devices must be analyzed and interpreted. Fortunately, advancements in technology have made ML a powerful tool for WQM. There are four main types of ML techniques used in WQM, namely supervised, unsupervised, reinforcement, and semi-supervised learning. Supervised learning is employed to classify WQ data based on specific parameters and detect anomalies or changes. Unsupervised learning identifies patterns or clusters within data to detect unusual WQ events or identify new patterns. Reinforcement learning optimizes treatment processes by providing feedback to control systems. Semi-supervised learning uses a combination of labeled and unlabeled data to perform classification or clustering. These ML techniques provide valuable insights into WQ trends and patterns and help in the development of effective WQM strategies.

**Fig. 2:**
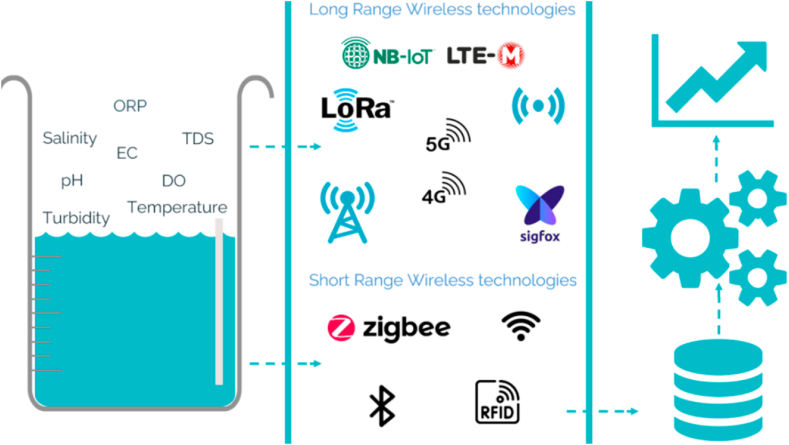
Enhancing WQ: Pollutants, IoT, and data analysis.

The integration of IoT devices with ML algorithms holds great promise for enhancing WQM by facilitating real-time data collection and analysis. This combination empowers the early detection and timely response to WQ issues, effectively safeguarding water resources and public health. Furthermore, IoT devices and ML algorithms can uncover the root causes of WQ problems through pattern recognition, shedding light on potential sources of contamination and the impact of human activities on WQ. This valuable information can serve as a foundation for policymakers and stakeholders to devise effective strategies for the preservation of water resources and the promotion of sustainable water management practices. Our analysis provides a comprehensive overview of the use of Internet of Things (IoT) and Machine Learning (ML) to WQM systems, demonstrating its flexibility in many domains. Our goal is to demonstrate the various uses of this integration, in many domains such as wastewater treatment and household WQM. by integrating IoT sensors and the power of ML algorithms for real-time monitoring, this paper highlights the ability to quickly identify pollutants and support well-informed decisions on purification systems, so guaranteeing the safety of drinking water. Moreover, our paper highlights its applicability in industrial settings, aiding in meeting regulatory standards for WQM, and in agricultural areas to improve water management practices. Through these varied applications, our goal is to underscore the transformative potential of the IoT and ML synergy in advancing WQM across different sectors (Fig. 2).

**Fig. 3:**
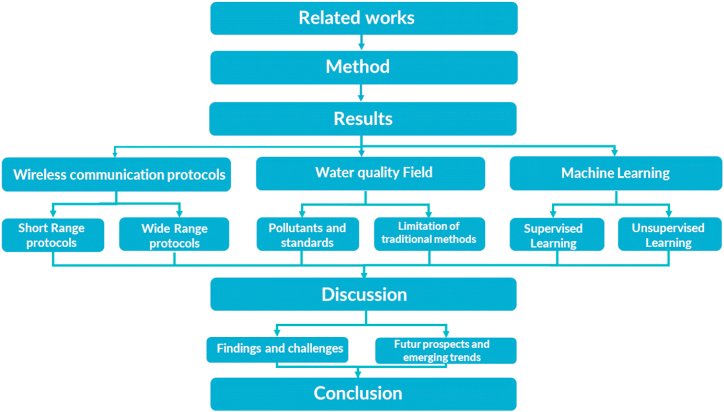
Mapping the review elements.

The paper is organized into several sections. Section 2 provides an overview of previous reviews on WQM and their significance. Section 3 explores the PRISMA approach used in the reviewing method. Section 4 focuses on WQM using wireless IoT technologies and ML techniques and. Section 5 discusses the challenges and future directions, and finally, Section 6 presents the conclusion (Fig. 3).

## 2 Related works

Many previous reviews and research papers have been dedicated to the exploration of WQM, encompassing a wide range of topics, methodologies, and technologies. These endeavors seek to deepen our understanding of WQ parameters and develop effective monitoring systems.

Jiang et al. [8] discussed quantitative design approaches for surface WQM networks, with an emphasis on cost-effective design. It identifies gaps and challenges in sampling frequency, WQ indicators, and pollution management. Behmel et al. [9] proposed a holistic strategy to support WQM programs. The paper addresses challenges in planning and optimization, emphasizing reliable WQ assessment, stakeholder involvement, and the integration of resources into an intelligent decision support system. Karydis and Kitsiou [10] focused on marine WQM principles, design, and data analysis procedures. The review highlights the use of statistics, simulation models, and multiple criteria analysis for compliance, trend detection, and policy assessment. The design of ground WQM networks is discussed by Loaiciga et al. [11], considering hydrogeologic and statistical approaches. Factors such as spatial scale, sampling objectives, and data requirements are taken into account.

In the context of IoT-based aquaculture, Prapti et al. [12] focused on WQM in fishponds. The review provides insights into research approaches, common parameters, and types of solutions provided. Banna et al. [13] evaluated emerging technologies for online WQM, with a specific focus on the need for low-cost sensors. The paper discusses the potential of data processing algorithms and envisions a future with widespread deployment of affordable WQ sensors. The potential of IoT technology for water management systems is explored by Singh and Ahmed [14], who conducts a comprehensive survey of existing IoT-based systems. The study discusses system architecture, and measurement parameters, and proposes future designs that integrate IoT and ML for predictive analytics. The potential of remote sensing, particularly deep learning (DL), in accurately estimating optically active parameters in WQM is emphasized by Sagan et al. [15]. The review emphasizes the need for improving remote sensing capabilities to enhance water resources management. Zhu et al. [16] explored the application of ML algorithms in evaluating WQ across different environments, including surface water, groundwater, drinking water, sewage, and seawater. They discussed challenges related to data acquisition, algorithm applicability, and interdisciplinary expertise. The review suggests future research directions to address these challenges. The review conducted by Dogo et al. [17] compared traditional ML and DL methods for detecting anomalies in WQ data. They favor DL approaches and propose a hybrid framework for further investigation in WQ anomaly detection. Ighalo et al. [18] performed a systematic literature analysis on artificial intelligence models in surface WQM. The paper identifies commonly used models such as the Adaptive Neuro-Fuzzy Inference System (ANFIS) and Artificial Neural Networks (ANN). The paper highlights knowledge gaps in the field and proposes future research directions. Ewuzie et al. [19] provided an overview of DL techniques in WQ modeling and prediction. The chapter covers topics like data acquisition, preprocessing, and model selection. It also discusses prospects in water research. Wagle et al. [20] examined the application of ML and remote sensing data for estimating WQ parameters. The integration of regression algorithms and ANN is explored, and the potential of real-time AI-enabled WQM systems is discussed.

Previous studies on WQM have focused on particular topics, like IoT technologies, ML approaches, and sensor techniques. Our study, however, adopts a different strategy. It focuses on combining ML techniques with wireless IoT technology to monitor WQ. It analyzes several IoT wireless technologies, including Bluetooth, Wi-Fi, Zigbee, RFID, LpWAN, and cellular networks. The investigation seeks to bridge knowledge gaps and investigate the benefits and prospective applications of ML algorithms with IoT wireless connectivity options. To streamline monitoring systems and foster the development of more advanced and effective water monitoring technology, the study underlines the significance of ML methods and IoT wireless technologies.

## 3 Method

**Fig. 4:**
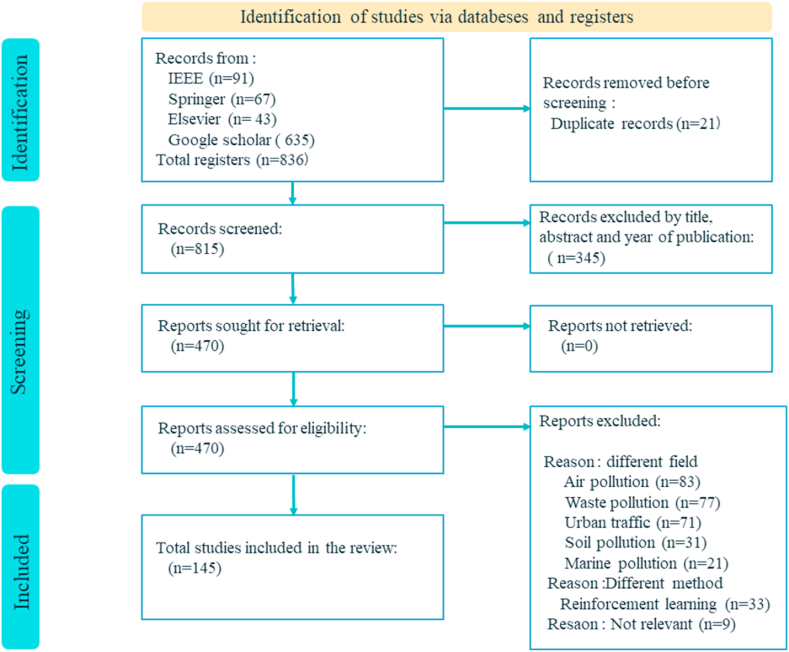
Prisma flowchart: Study selection process.

Our systematic review used the Preferred Reporting Items for Systematic Reviews and Meta-Analysis (PRISMA) procedure [21]. PRISMA offers an organized and transparent approach to thorough literature reviews, hence improving rigor and repeatability. It entails developing research topics, choosing studies, evaluating quality, and synthesizing results. Using PRISMA, we collected current publications on IoT, ML, and WQ, with an emphasis on environmental concerns in urban areas. For this purpose, a comprehensive set of keywords and key phrases were meticulously chosen, aligning with the research objectives and scope (WQ, Urban environment, Environmental monitoring, ML, IoT, Wi-Fi, Bluetooth, Zigbee, Predictive modeling, etc.) Specialized databases are searched using these keywords. This produced 141 relevant articles for our comprehensive analysis (Fig. 4).

● **database collection**

The process of conducting the review was initiated by undertaking a collection of academic articles that focused on the intersection of ML, IoT, and WQ. To ensure a comprehensive scope, renowned databases including but not limited to Google Scholar, IEEE, Springer, and Elsevier were meticulously leveraged. These platforms facilitated access to a wide-ranging assortment of journal articles, thereby enriching the diversity of the gathered literature. In line to keep the review both pertinent and up-to-date, particular emphasis was placed on sourcing recent publications that encapsulated the latest advancements and insights in this evolving field.

● **initial data collection**

From the original compilation of approximately 504 articles, the subsequent phase encompassed a preliminary screening procedure designed to discern articles harmonizing with the designated research focus. This preliminary screening primarily scrutinized titles and abstracts, thereby singling out articles that were in alignment. The emphasis at this stage was distinctly placed on articles that delved into the realm of applying ML techniques, encompassing supervised, unsupervised, reinforcement, and semi-supervised approaches, as well as delving into the domain of IoT wireless technologies. The overarching aim was to identify articles that distinctly tackled the intricate web of environmental challenges through the symbiotic integration of ML methodologies and IoT wireless technologies. Furthermore, a temporal constraint was imposed, confining the search within recent years. This meticulous time frame limitation was integral to ensuring the assimilation of the most contemporaneous advancements, insights, and trends that have unfolded in this dynamic field.

● **preliminary screening**

Among the chosen articles, there existed a dedicated commitment to accentuating the pivotal theme of WQ. Articles that distinctly showcased a lucid focus on various dimensions of WQ, including but not limited to environmental sustainability, urban livability, efficiency in transportation systems, and optimization of resources, were accorded a preferential position for further in-depth analysis. Within the framework of the review, an intricate exploration unfolded, as the selected articles underwent an exhaustive scrutiny. This scrutiny entailed a thorough and comprehensive assessment of the meticulously employed supervised and unsupervised ML methodologies. Simultaneously, equal attention was directed towards the examination of short-range and wide-range IoT connectivity, with a targeted spotlight on specific technologies: Wi-Fi, Bluetooth, Zigbee, cellular networks, RFID, and Low-Power Wide-Area Networks (LpWAN).

The evaluation process ventured into the intricate fabric of these methodologies and technologies, dissecting aspects such as sensing techniques, the discernment behind algorithm choices, techniques for preprocessing data, the intricacies of feature engineering, the robustness of model evaluation, and the seamless integration of domain-specific knowledge. By meticulously delving into these facets, a comprehensive understanding of the efficacy and resilience of the adopted approaches emerged, shedding light on their potential in addressing the multifaceted challenges related to WQ and its interconnected domains.

● **Selection In Depth Assessment And Retrieval**

In the curation of the definitive articles for this comprehensive review, an exacting approach was embraced to ensure the assimilation of top-tier, indexed research that embodies a rich diversity in both IoT wireless technologies and supervised and unsupervised learning methodologies. This meticulous approach guarantees a judicious selection that encapsulates a broad spectrum of cutting-edge approaches.

The chosen articles elegantly traverse an extensive spectrum of methodologies, encompassing classification, regression, DL, and innovative hybrid models. This multifaceted collection affords a profound exploration into their manifold applications within the intricate landscape of WQ research. Furthermore, a particular emphasis was placed on encompassing a gamut of pollutants, including but not limited to pH, Dissolved Oxygen (DO), Turbidity (TUR):, Total Dissolved Solids (TDS), Electrical conductivity (EC), Temperature (Temp), Chemical oxygen demand (COD), Biological Oxygen Demand (BOD), Oxidation Reduction Potential (ORP) and Water Quality Index (WQI). This deliberate inclusion of diverse pollutants has bestowed an unparalleled vantage point, thereby revealing multifaceted insights into the intricate tapestry of challenges entailed in the arena of water pollution management.

The rigors of this stringent and meticulous selection process culminated in the assembly of a compendium encompassing 80 articles within the IoT domain and an additional 61 articles within the ML sphere. Collectively, these articles serve as the bedrock upon which a profound and discerning analysis is built at the juncture of supervised learning methodologies and the intricate interplay of diverse pollutants in the realm of WQ research.

● **synthesis and presentation**

The synthesis incorporates this article into a dedicated section, comprising three subsections. The first subsection delves into the discussion of WQ, elucidating its significance. The subsequent subsection addresses Wireless IoT techniques, focusing on both short-range and wide-range applications. The final subsection examines ML techniques, encompassing both supervised and unsupervised methods. This amalgamation of reviewed articles culminates in a coherent and meticulously organized narrative. The synthesis initiates with an introductory segment that underscores the significance of integrating IoT and ML in the domain of WQ research. It then navigates through diverse IoT and ML strategies employed to combat challenges related to WQ, while also analyzing their implications on urban quality outcomes. Emphasis is placed on spotlighting pivotal studies and their potential to shape forthcoming research and urban planning strategies.

## 4 Results

### 4.1 Water quality field

71 percent of the surface of the Earth is covered by water, with freshwater making up only 2.5 percent of that area [22]. Water is a vital component of human life that is utilized in important spheres of society like industry, agriculture, and households. Water quality and quantity are being threatened by misuse as a result of the increasing population, economic and industrial growth.

The two forms of water resources are groundwater and surface water. 60 percent of the world's freshwater requirements are met by groundwater [23],30 percent of the freshwater on Earth is found in groundwater, while the remaining 69 percent is found in rivers, lakes, glaciers, ice sheets, icebergs, and ice sheets [24]. The identification of groundwater pollution is a big concern, because it is more challenging than the detection of surface water contamination.

The World Health Organization (WHO) has declared a state of emergency over water availability, emphasizing that half of the world's population will live in water-stressed areas and recognizing the human right to a safe, adequate, and uninterrupted supply of water [25]. WHO also declared that nearly two billion people worldwide consume polluted water [26], their health is negatively impacted by contaminated water, bad sanitation, and unhygienic living conditions. Safe water might save 860,000 cases of malnutrition, 500,000 cases of malaria, and 1.4 million cases of diarrhea in children [27].

● **pollutants and standards of water quality**

**Fig. 5:**
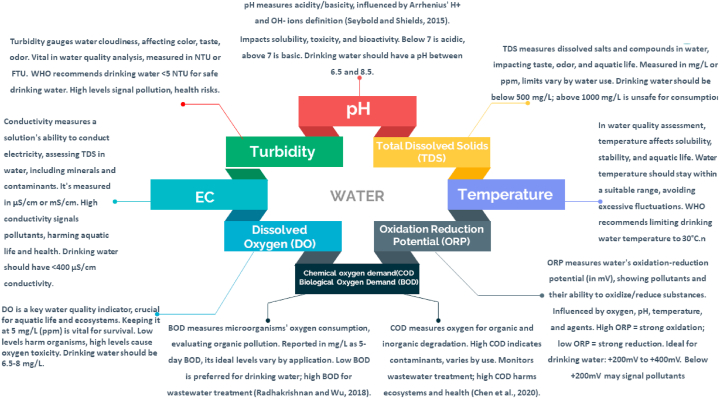
Key parameters for water quality assessment.

In addition to scarcity, water contamination is a serious problem, With the help of the thresholds of contaminants, WQ can be characterized, There are around 101 key factors that are utilized to control WQ, according to the Environmental Protection Agency [22], WHO has defined and set the ranges and limits of drinking water parameters [28], these are the key parameters of WQ (Fig. 5).

Salinity, Heavy metals (HMs) Chlorine (Cl^−^), Total Phosphorus (TP), Magnesium (Mg), Calcium (Ca^2+^), Sodium (Na), Potassium(K), Fluoride(F^−^) Nitrate (NO^3−^) and other substances also have an impact on WQ. These characteristics become detrimental to water and a host of health problems when they surpass the standards ([23,24,29,30]).

A wide range of commercially accessible WQ sensors are designed to measure various water characteristics. For instance, pH sensors (DPD1R1-WDMP), which are essential for determining the acidity or alkalinity of water by measuring the concentration of hydrogen ions on a scale from 0 to 14. Oxygen dissolved in water is measured by DO sensors (YCS-2000 DO sensor). Turbidity sensors (LXV423.99.10100) employ light-scattering techniques to measure the cloudiness induced by suspended solids in order to calculate the amount of suspended solids. Additional sensors include conductivity sensors (D3725E2T-WDMP) and ORP sensors (DRD1R5-WDMP), which measure the EC of water and provide information on the concentrations of ions or dissolved salts. Temperature sensors (DPD1R1-WDMP and Pt1000 temperature sensor) track the temperature of the water, which is a critical factor influencing many aspects of WQ. Every sensor has distinct sensitivities and measuring ranges. ([31,32]).

To efficiently describe WQ, Horton proposes the water quality index (WQI) [33] (Fig. 2). WQI is a numerical index that provides a summary measure of the overall WQ of a particular water body. Different methods and approaches are employed to calculate the WQI, depending on the assessment objective, data accessibility, and local regulations. Among the commonly used methods are the Canadian Council of Ministers of the Environment (CCME) WQI, National Sanitation Foundation (NSF) WQI and Oregon Water Quality Index (OWQI). These methods take into account various parameters, assign weights to them, and integrate the scores to derive the overall WQ [34].

**Fig. 6:**
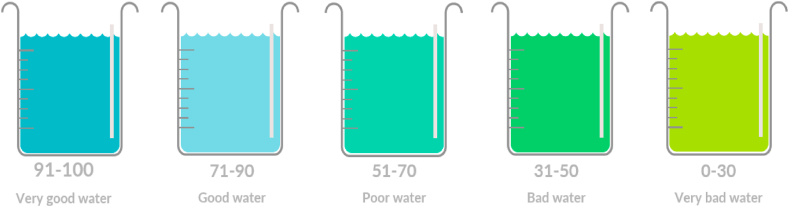
Ranges of WQI

The WQI ranges from 0 to 100, where a higher WQI value indicates better WQ (Fig. 6). The index can be used to compare the WQ of different water bodies, monitor WQ over time and identify areas where WQ needs improvement. The WQI is a useful tool for water resource management and decision-making, as it provides a simple and straightforward way to communicate complex WQ data to stakeholders the parameters influencing WQI the most are pH, BOD, COD, TDS, DO, and ammoniacal nitrogen [35].

● **limitation of traditional methods in WQM**

Traditional WQM methods are associated with several limitations that impede their effectiveness. These constraints stem from factors such as the methodology used for sample collection, the time required for laboratory analysis, and the restricted geographic coverage. It is crucial to acknowledge and understand these limitations in order to expand and enhance our WQM techniques effectively.

One of these limitations is the limited sample frequency. Traditional approaches frequently rely on manual sampling, which can only produce a certain number of data points over time. An insufficient understanding of changes in WQ may result from this occasional sampling, which may not adequately capture the dynamic nature of WQ measures. For conventional water-quality monitoring programs, the significant temporal variability in pollutant concentrations presents a difficulty. Inaccurate maximum and average concentration estimates may result from infrequent grab sampling techniques missing instances with high pollutant concentrations. This makes it challenging to precisely capture the spatial diversity of WQ indicators [36].

Lack of Real-time and reliable Data is another issue, traditional approaches use batch analyses of water samples, which can take hours or even days to complete and are only available for a limited amount of time. This implies that transient changes in WQ, such as pollution spikes or tidal oscillations, may be overlooked or misreported. In addition, the time it takes to get results limits the real-time data that is needed for prompt decision-making and efficient pollution management. This limits our ability to swiftly detect and respond to abrupt changes in WQ. Traditional methods often focus on a small set of predetermined WQ criteria. This sparse parameter coverage can overlook newly emerging contaminants or other crucial markers of WQ. The amount of factors that present sensors can measure is constrained, and they frequently fall short of users ‘expectations [37]. Given the variety of hazards, groundwater monitoring, for instance, should give preference to site-specific parameters to identify WQ issues [38].

Due to the necessity of physically collecting samples from specific monitoring locations, traditional approaches sometimes suffer from geographical restrictions. This method falls short of offering a thorough knowledge of how WQ varies over bigger water bodies or watershed areas. This limitation may cause localized sources of contamination to go undetected or may result in inaccurate spatial patterns and gradients of WQ measurements being recorded. Investigating spatial WQ trends and fluctuations is difficult, especially in large water bodies. Entire water bodies may be difficult or impossible to monitor, forecast, or manage, especially when topographic restrictions apply. Due to errors in both field sampling and laboratory analysis, the precision and accuracy of the in-situ data acquired may be in doubt [39].

The traditional approaches are labor-intensive, expensive, and time-consuming; they frequently involve fieldwork, sample collection, transportation, trained personnel, laboratory analysis, and sampling equipment. Real-time monitoring and rapid action are hampered by these labor- and time-intensive processes, which cause delayed results. Traditional techniques of WQM might incur large costs for equipment, labor, and laboratories. Particularly in locations with limited resources, this high cost may limit the frequency and scope of monitoring. Traditional methods for measuring WQ indicators involve costly and time-consuming laboratory analysis, which makes it challenging to identify contaminants quickly and understand their effects [40].

Invasive sampling is another problem with traditional WQM methods. These techniques frequently involve physically taking water samples at certain locations, which could harm the environment. The reliability and caliber of the data acquired are impacted by the collection, transportation, and storage of samples since they increase the number of potential sources of contamination and impede natural processes. Early warning systems that use traditional event detection techniques to identify contamination events frequently experience large error rates. These techniques mostly depend on the variance between observed and predicted sensor responses, which can result in inaccurate detection [41].

### 4.2 Wireless communication protocols in WQM

Traditional WQM methods have been time-consuming, costly, and often result in inadequate data collection. However, with the advent of IoT wireless technologies, it has become possible to monitor WQ in real time, providing accurate and reliable data on the health of water resources.

The introduction of IoT technology has transformed the way WQ data is gathered. IoT enables continuous monitoring and transmission of various WQ parameters by deploying a network of interconnected sensors. These sensors, strategically deployed throughout water bodies, pipelines, treatment plants, and distribution networks, can measure a broad spectrum of parameters, encompassing temperature, turbidity, pH levels, and the presence of contaminants such as chemicals or heavy metals. These IoT-enabled sensors collect data in real-time, allowing for the early detection of abnormalities or departures from established standards. Traditional centralized systems and manual procedures may be improved with the use of wireless sensor networks, resulting in decentralized smart WQM systems that are adaptable enough to accommodate the diverse and dynamic water distribution networks of cities [42].

Moreover, IoT technology makes it easier to combine and send the data that these sensors gather to a cloud-based platform or centralized system over the internet. The complete gathering of data from several dispersed sensors is made possible by the devices' continuous connection with each other. Using web-based dashboards or mobile applications, stakeholders—such as environmental agencies, water treatment authorities, and researchers—can access this data remotely and in real-time. aiding in efficient water management and decision-making processes [43]. The IoT capacity to store WQ data opens up the possibility of applying advanced analytics techniques to uncover patterns, trends, correlations, and potential risks. This information empowers predictive maintenance strategies, enabling the early detection of anomalies and optimizing resource allocation for efficient water management. By integrating IoT-collected data into decision-making systems, water treatment processes, distribution, and resource allocation can be further optimized, leading to improved operational efficiency and higher WQ outcomes.

IoT wireless technologies, including short-range technologies such as Wi-Fi, Zigbee, RFID, and Bluetooth, and long-range technologies like LpWAN and Cellular networks, have transformed the way we monitor WQ. These technologies enable seamless communication between sensors and devices, facilitating the collection and transmission of data to monitoring systems. This real-time data collection allows for more efficient and effective WQM, resulting in improved resource management and preservation.

RFID, Bluetooth, Zigbee, and Wi-Fi have all played crucial roles in the development of wireless communication technologies. RFID's origins can be traced back to its use in identifying aircraft during World War II [44], and later it found its way into commercial applications such as access control systems, supply chain management, inventory tracking, and contactless payment systems. Bluetooth, which emerged in the 1990s [45], revolutionized the way devices connect by eliminating the need for cables and enabling wireless communication between mobile phones, computers, and peripherals. Zigbee, introduced in the early 2000s [46], focused on low-power communication for home automation and sensor networks, providing the ability to create mesh networks for seamless device integration. Wi-Fi, introduced in 1997 [47], brought wireless internet connectivity to homes, businesses, and public spaces, offering data exchange capabilities within a specific range. Over the years, Wi-Fi has seen remarkable advancements in terms of speed, range, and security protocols, fueling the proliferation of smart devices and applications. Today, Wi-Fi has become an integral part of our daily lives, allowing us to stay connected and empowering the seamless operation of a wide range of smart devices.

Cellular networks have a rich history that goes back to the late 20th century. It all began with the first-generation (1G) analog networks in the 1980s. Then, in the late 1980s, second-generation (2G) networks were introduced, bringing digital voice transmission and SMS capabilities. As we entered the new millennium, third-generation (3G) networks came into play around 2000, making mobile internet, video calling, and multimedia messaging widely accessible. Later, in the late 2000s, fourth-generation (4G) networks emerged, offering faster speeds and fueling the growth of mobile applications. More recently, fifth-generation (5G) networks have taken the spotlight, promising blazing-fast speeds and enabling transformative technologies like IoT [48]. Alongside the evolution of cellular networks, LpWAN technologies have emerged to cater to specific connectivity requirements. LpWAN have a relatively recent history starting from the early 2010s. At that time, there was a growing demand for connectivity solutions that could provide long-range and low-power connections for IoT applications. Companies like Sigfox and LoRaWAN came up with their own technologies using unlicensed spectrum bands to extend coverage. In 2016, the standardization efforts by 3GPP led to the development of the NB-IoT standard, which enabled LpWAN capabilities on licensed spectrum bands [49]. Since then, LpWAN technologies have continued to evolve and find their way into various industries. Ongoing advancements and deployments indicate that LpWAN will have an important role in connecting a wide range of IoT devices in the future. Fig. 8 represents a chronological overview of the advancements in wireless IoT technologies. This timeline offers a visual journey through the evolution of IoT communication protocols.

**Fig. 7:**
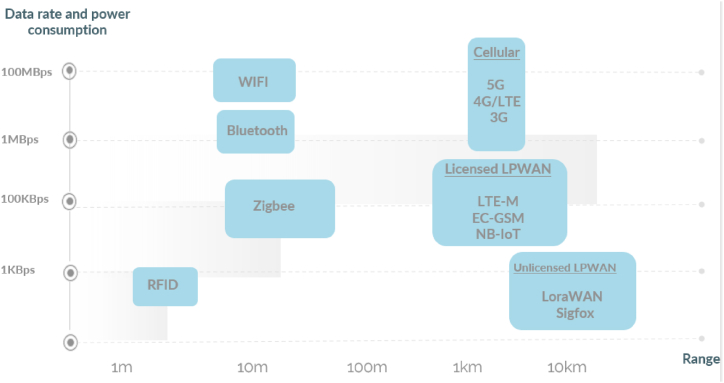
IoT wireless technologies range and bandwidth.

The selection of an appropriate IoT wireless technology for WQM entails considering multiple factors such as the specific application requirements, environmental conditions, and budgetary limitations. Due to the diverse range of wireless technologies available for this purpose, selecting the most suitable technology for a given application can be a challenging task.To facilitate the technology selection process, Fig. 7 provides a comprehensive representation of the bandwidth and range characteristics associated with various wireless technologies, such as Wi-Fi, Zigbee, RFID, Bluetooth, LpWAN, and Cellular networks, typically employed in WQM. By evaluating these features, it becomes easier to identify the most appropriate technology for a specific application.

**Fig. 8:**
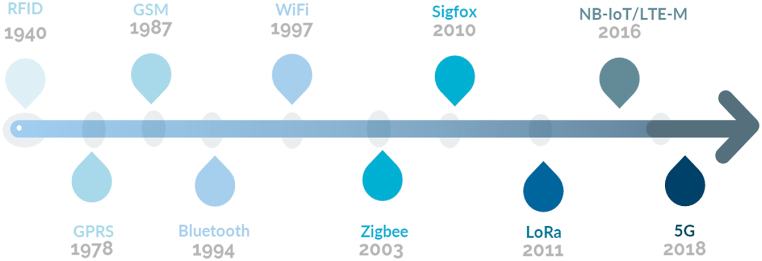
Timeline: Evolution of IoT wireless technologies.

#### 4.2.1 Short-range wireless connectivity applications

Short-range wireless technologies, such as Wi-Fi, Zigbee, RFID, and Bluetooth, are designed to provide wireless communication over a limited distance, typically within a few hundred meters. These technologies are ideal for applications where the devices are in close proximity to each other, such as in a smart home or building automation environment. One of the significant advantages of short-range technologies is their low power consumption, which extends the battery life of connected devices. These technologies are also relatively low cost, making them accessible for a wide range of applications. In addition, they offer the ability to form mesh networks, allowing devices to relay information to each other for increased reliability.

● **Bluetooth technology for WQM**

IoT Bluetooth technology is a highly popular option for many IoT applications, primarily due to its low power consumption and ease of use. This technology enables devices to exchange data over short distances without requiring complex wiring or internet connectivity. Moreover, it is also an ideal solution for WQM and management due to its cost-effectiveness. By integrating Bluetooth technology into sensors, WQ parameters like pH, DO, and Temp can be measured, and the data can be transmitted to a central database or monitoring system for real-time analysis.

Several studies have utilized Bluetooth technology for data transmission and analysis. For instance, in a study conducted by Ijaradar and Chatterjee [50], Bluetooth-enabled sensors were employed to measure WQ parameters such as pH, TUR, and Temp. The collected data was then transmitted to a cloud-based database. Similarly, Jha [51] evaluated groundwater using Bluetooth-enabled sensors and implemented a system to send data to mobile devices via GSM and Bluetooth in case the parameters exceeded standard limits. This approach facilitated timely detection of potential issues and enabled effective WQM.

In the realm of mobile applications, Bluetooth communication has played a significant role in WQ assessment. The study conducted by Srivastava et al. [52] developed an Android app that utilized Bluetooth connectivity to display real-time WQ characteristics and upload it to the cloud. Mabrouki et al. [53] developed an app with an incorporated predictive model to estimate WQI, This approach enabled users to conveniently access real-time WQ information, Bluetooth was employed to transmit water parameters, such as Temp, TUR, EC, and DO, to smart objects like a smartwatch. Additionally, Jindal et al. [54] proposed a smartphone-based Bluetooth framework that employed multi-parametric sensors to determine the physical characteristics of sewage water, including pH, and EC. The collected data proved to be valuable for WQM and treatment processes. Additionally, Jo et al. [55] proposed an innovative solution by developing an USV controlled via Bluetooth through an Android app. This USV allowed users to view sensor and GPS data, including TUR, and pH near the water surface. Such advancements in Bluetooth-based technologies contribute to improved WQM and management, offering efficient and convenient solutions for data collection and analysis.

● **RFID-based systems for enhanced WQM**

RFID technology is an emerging field that uses radio waves for the transfer of data between a reader and a tag, enabling the identification and tracking of an object. In the realm of WQM, RFID technology is being utilized to enhance the effectiveness and accuracy of monitoring systems. Instead of using conventional monitoring methods that necessitate manual sampling and laboratory testing, RFID-based systems utilize sensors affixed to RFID tags to collect real-time data, reducing the requirement for manual intervention.

Several research papers have proposed innovative wireless monitoring systems for waterways that utilize portable sensor nodes integrated with RFID technology. These systems enable the measurement and transmission of various WQ parameters, including pH, Temp, DO, and TUR. The collected data is then processed and analyzed at a centralized location, facilitating effective monitoring and analysis of WQ [56]. In one study, researchers described the use of low-cost analog sensors, 1-wire networking, and passive tags to ensure compatibility with IEEE 1451.4 standards [57]. Another research introduced a novel wireless passive sensing platform that combined inkjet printing, microfluidics, and RFID technology to monitor WQ parameters [58]. These advancements demonstrate the potential of RFID technology in WQM applications.

Furthermore, authors have explored the integration of RFID technology with other sensing techniques to enhance the reliability and efficiency of WQ measurements. For instance, one study proposed the use of an analog pH sensor with IP-based communication and RFID technology, offering improved pH measurement capabilities [59]. Additionally, Cook et al. [60] presented a real-world implementation of RFID-based sensors for monitoring various environmental parameters. This study highlighted the potential of RFID-based sensors in monitoring applications and provided valuable insights into their implementation and deployment in real-world scenarios. These research findings contribute to the development of advanced monitoring systems that leverage RFID technology, enabling more efficient and accurate assessment of WQ in different contexts.

● **Wi-Fi networks for real-time data collection**

In some studies, existing Wi-Fi networks were utilized for real-time data collection. For instance, Chen and Han [61] made use of the “Bristol Is Open” infrastructure to collect real-time WQ data using a multiparameter sonde. The Wi-Fi transceiver module has also played a crucial role in transferring WQ data to cloud-based servers for further processing and analysis. Parameswari and Moses [62] utilized the Wi-Fi transceiver module in conjunction with water sensors to transmit data to a cloud node server via an Arduino microcontroller. This cloud-based approach facilitated centralized data storage, analysis, and access.

In certain cases, Wi-Fi connectivity has been used to enable remote monitoring and control of WQ assessment systems. For example, Melo et al. [63] developed a robotic airboat equipped with WQ sensors, which transmitted data through a Wi-Fi connection to a base station. This setup allowed users to monitor the vehicle and plan routes based on the WQ information. Moreover, the combination of Wi-Fi and other wireless communication technologies has been explored for WQM applications. Jindal et al. [54] utilized multi-parametric sensors to monitor sewage WQ and connected them to a smartphone or laptop using both Bluetooth and Wi-Fi technologies, enabling versatile data transmission options.

● **Zigbee mesh networks for scalable WQM**

Zigbee is a wireless communication technology that has gained popularity in recent years for its low-power, low-cost, and reliable capabilities. It uses mesh network architecture to create large and scalable networks, making it an ideal technology for a range of applications, including WQM. Traditional WQM systems typically involve manual sampling and laboratory analysis, which may be time-consuming and prone to errors. However, Zigbee-based WQM systems use sensors connected to a Zigbee network to collect real-time data on parameters such as pH, Temp, and DO levels, reducing the need for manual intervention and providing real-time data for early problem detection.

Zigbee technology offers significant benefits in WQM systems. In a study by Jha (2020) [51], microcontrollers and sensors for various parameters are connected through Zigbee/Wi-Fi technology to monitor WQ in tanks and wireless sensor zones. This enables the creation of large-scale monitoring networks that provide valuable insights into WQ over time. The study also includes alerts to promptly address unsafe drinking water conditions and prevent degradation. Another study by Usha Kumari et al. [64] utilizes Zigbee technology to monitor WQ in a specific area, employing sensors for pH, TUR, TDS, and Temp. The integration of solar power modules and GSM allows for autonomous control, facilitating continuous monitoring without manual intervention. Real-time monitoring enables early problem detection, enabling timely intervention to prevent further deterioration. Additionally, Al-Dahoud et al. [65] implements a hierarchical sensor network to lower costs and increase sensor node deployment density. Each node is equipped with low-power panels, two solar collectors, and utilizes low Zigbee radio power for data transmission. The microcontroller processes the sensor data before sending selected information to the gateway via an Xbee module. This approach significantly reduces the cost of sensor node deployment, making it more accessible for diverse applications.

#### 4.2.2 Wide area connectivity applications

● **Cellular Networks: Enabling Real-time WQM with Reliability and Cost-effectiveness**

In the context of WQM, cellular networks provide a reliable and cost-effective option for transmitting real-time data remotely to servers, enabling efficient WQM. The use of cellular networks, including 2G, 3G, 4G, and 5G, has been explored in various studies, depending on the specific requirements of the application. For instance, Ionel et al. [66] proposed an experimental virtual instrumentation system based on General Packet Radio Service (GPRS) for the automatic measurement and assessment of nitrates (NO3-), copper (Cu++), and chloride (Cl-) levels in water. The system enabled users to access data using a mobile device, and automated SMS and email notifications were sent when monitored metrics exceeded predetermined levels.

Similarly, Adamo et al. [67] developed an in situ and continuous monitoring system for seawater parameters, such as Temp, EC, TUR, and chlorophyll levels, using GPRS and GPS modules, along with smart sensors. The wireless communication facilitated real-time probe tracking and data interpolation. Saravanan et al. [68] proposed a Supervisory Control and Data Acquisition (SCADA) system that utilized 4G/5G technologies to transfer data and GSM connection for continuous monitoring of WQ parameters across multiple stations. The SCADA system recorded real-time and accurate flow, Temp, color, and TUR readings from the sensors utilizing GSM connectivity. The automated system has been implemented within the Tirunelveli Corporation (a metropolitan area in the state of Tamil Nadu, India) to collect sensor data automatically, including information from pressure, pH, level, and energy sensors. This technological method demonstrates versatility in various global settings, providing effective WQM in diverse geographical areas and environments. Esakki et al. [69] developed an unmanned aerial vehicle (UAV)-based monitoring system that used a 4G-LTE network for data transmission to the ground station through Firebase cloud services. The system facilitated real-time measurements of WQ parameters, such as pH, DO, TUR, and EC, and enabled remote and continuous monitoring of water bodies.

● **LpWAN: energy-efficient and extended-range technologies for WQM**

Numerous investigations have examined the application of LpWAN in WQM systems. For instance, Huan et al. [70] described an NB-IoT-based system that continuously transmits real-time data on environmental factors, including Temp, pH, DO, and other parameters, in aquaculture ponds. Similarly, Elijah et al. [35] uses NB-IoT and LoRa to monitor various WQ parameters, like pH, AN, SS, and DO of Malaysian rivers, via UAV surveillance.

Moreover, the use of LoRa technology is also prevalent in WQM systems, as demonstrated in many studies. Madeo et al. [71] employed LoRa technology for data transmission in an USV to collect WQ data, such as Temp, pH, ORP, DO, and salinity in rivers, lakes, and oceans. Meanwhile, Simitha and Subodh Raj [72] utilized LoRaWAN to remotely monitor WQ parameters, including pH, TUR, Temp, and DO. The system's energy efficiency is improved by the low energy consumption of LoRaWAN, allowing for an extended battery life for sensor nodes.

Additionally, Sigfox is chosen as the communication protocol for WQM by Di Gennaro et al. [73], and Boccadoro et al. [74]. Sigfox's IoT platform provides secure communication and enables extensive transmission of WQ measurements. For example, Boccadoro et al. [74] used Sigfox communication technology to remotely relay measurements and forecast WQ metrics, such as pH, EC, oxygen, and Temp, via the WaterS IoT WQ prediction system. Silva [75] developped a web-based system for WQM, which collects various WQ information using a WiMax network and a local Zigbee network. DO, EC, Temp, pH, and ORP sensors are among the other monitored parameters. The data from sensor nodes is transmitted via WiMax to the web server using the Zigbee gateway.

These studies demonstrate that while there is a lot of research being done on smart WQM, relatively few initiatives get full and ongoing government backing. Many natural disasters over the past 10 years, such as earthquakes, tsunamis, and droughts, have compelled governments and water utilities to create proactive measures in order to ensure water security. Diverse global approaches have been employed to protect the quantity and caliber of water required for urban areas. Few governments—like Singapore's Water Supply Network Department (PUB) and Australia's South East Queensland (SEQ) Water Grid—have been successful in developing innovative water management systems. The South East Queensland (SEQ) Water Grid and the Water Supply Network Department (PUB) in Singapore have implemented comprehensive water strategies to improve WQM. The SEQ Water Grid uses climate-resilient water sources like desalination and purified recycled water, as well as rainwater, to ensure water security. Meanwhile, the PUB collects rain and storm water, treats it, and integrates it into the water network to maintain WQ. Both systems have integrated information and communication technology (ICT) into their water management systems to improve WQ. The SEQ Water Grid uses standardized sensor systems for WQ, quantity, and pressure, while the PUB plans to spend several billion USD on projects to establish and extend their smart water management system, integrating ICT-based solutions for WQM and management. These initiatives highlight the importance of integrating ICT in water management systems for sustainable water resources [76].

**Fig. 9:**
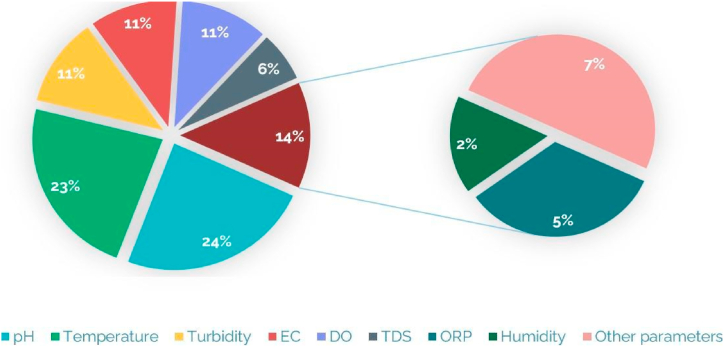
Pollutant monitoring: Percentage breakdown of monitored contaminants.

**Fig. 10:**
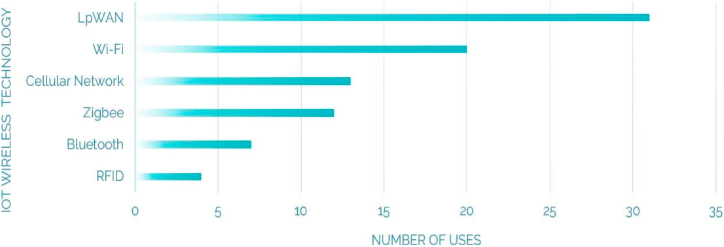
IoT wireless technologies: Percentage breakdown of utilization in WQM

Table 1 provides an overview of IoT wireless technologies employed in WQM, highlighting the parameters being monitored. Fig. 9 presents a graphical representation of the percentage breakdown of monitored contaminants, showcasing the distribution of pollutants in WQ assessment. This figure provides a visual understanding of the relative importance and prevalence of different contaminants Fig. 10 illustrates the percentage breakdown of IoT wireless technologies utilized in WQM, shedding light on their utilization and popularity in the field.

**Table 1:** IoT wireless technologies for WQM: A table Overview.

Reference	Year	Communication	Monitored parameters
Rahu, Mushtaque Ahmed et al. [77]	2024	WiFi	pH, TDS, TUR
González, Luis et al. [78]	2024	LoRaWAN	Temp, DO, ORP, and pH
Jamroen et al. [79]	2023	NB-IoT	DO, pH, Temp, TUR and salinity
Mansor and Abdul Latiff [80]	2023	LoRaWAN/GSM	pH, Temp and TUR
Gupta et al. [81]	2023	Wi-Fi/Bluetooth	DO, ORP, DS, Temp, EC and pH.
Razman et al. [82]	2023	Wi-Fi	Temp, pH, ORP, EC and TUR
Rao et al. [83]	2023	GSM	Temp, pH and TUR
Murugan et al. [84]	2023	Wi-Fi	Temp, pH and DO
Li et al. [85]	2022	LoRaWAN,5G	Temp, humidity and pH
Akhter et al. [86]	2022	LoRaWAN	Temp, nitrate, phosphate and pH
Boccadoro et al. [74]	2022	Sigfox	pH, EC, oxygenation and Temp.
Hartono et al. [87]	2022	LoRaWAN	pH and oxygen
Savel et al. [88]	2022	LoRaWAN	pH, Temp, TUR and EC
Sendra et al. [89]	2022	LoRaWAN	Temp, TUR and relative humidity(RH)
Fonseca-Campos et al. [90]	2022	Wi-Fi	ORP, pH, TDS, TUR, Temp, EC and DO
Abidin et al. [91]	2022	Wi-Fi	TDS and pH
Zeng et al. [92]	2022	LpWAN	pH, DO, K, permanganate and AN
Baghel et al. [93]	2022	LoRaWAN	TDS, Ec, Temp and pH
Wang and Lv [94]	2022	NB-IoT	Temp, pH, TDS and ORP
Lloret et al. [95]	2021	LoRaWAN,Wi-Fi	TUR, salinity and oil
Mubarak et al. [96]	2021	Bluetooth	Temp, pH and TUR
Tahatahir et al. [97]	2021	LoRaWAN	pH, TUR, TDS, DO and Temp
Liloja et al. [98]	2021	LoRaWAN	pH, TUR, Temp and EC.
Nurwarsito and Christian [99]	2021	LoRaWAN	pH, Temp and TUR.
Raditya et al. [100]	2021	LoRaWAN	Temp, pH and TUR.
Lee et al. [101]	2021	NB-IoT	Alga.
Philip and Singh [102]	2021	LoRaWAN	pH, TUR and Temp.
Islam et al. [25]	2021	Wi-Fi	Temp, pH and TDS.
Haque et al. [103]	2021	Zigbee	EC, DO, TUR, pH and Temp
Hsieh et al. [104]	2020	LoRaWAN	Temp, pH, EC and TUR
Jha [51]	2020	Bluetooth, GSM, Zigbee, Wi-Fi	TUR, TDS, EC, BOD, nitrate and fecal coliform, pH.
Mabrouki et al. [53]	2020	Bluetooth, Wi-Fi	Temp, pH, TUR, EC and DO.
Huan et al. [70]	2020	NB-Iot	Temp,pH and DO
Alset et al. [105]	2020	LoRaWAN	Temp, TUR and pH.
Ann Roseela et al. [106]	2021	Wi-Fi	Temp and TUR.
Al-Dahoud et al. [65]	2020	Zigbee	Temp, humidity, PH, DO, EC and TUR
Usha Kumari et al. [64]	2020	Zigbee	pH, TUR and Temp.
Jerom B. et al. [22]	2020	Wi-Fi	Temp, Humidity,CO2, pH and DO.
Sarnin et al. [107]	2020	GSM	Temp and humidity.
Arvind et al. [108]	2020	Wi-Fi	pH, TDS, TUR and Temp.
Bai et al. [109]	2020	Zigbee/GSM	pH and Temp
Yunfeng and Tianpei [110]	2019	NB-IoT	DO
Jo et al. [55]	2019	Bluetooth	Temp, TUR and pH
Simitha and Subodh Raj [72]	2019	LoRaWAN	pH, TUR, Temp and DO
Madeo et al. [71]	2020	LoRaWAN	Temp, pH, Salinity, ORP and DO.
Chowdury et al. [111]	2019	Wi-Fi	TUR, pH and Temp
Melo et al. [63]	2019	Wi-Fi	pH, EC, DO, ORP, TUR, Temp and chlorophyll.
Aggarwal et al. [112]	2019	Wi-Fi	EC, pH, DO, Color and TUR.
Budiarti et al. [113]	2019	4G	IP
Wu and Khan [114]	2019	LoRaWAN	DO, pH, TUR and Temp.
Ngom et al. [115]	2019	LoRaWAN	pH, EC, ORP and Temp.
Manoharan and Rathinasabapathy [116]	2018	LoRaWAN	TUR, pH, EC,DO, Nitrate, Arsenic and Fluoride.
Ijaradar and Chatterjee [50]		Bluetooth	pH, TUR and Temp.
Srivastava et al. [52]	2018	Bluetooth	Temp, pH and TDS.
Saravanan et al. [68]	2018	4G/5G/GSM	Temp, color and TUR.
Esakki et al. [69]	2018	4G-LTE	pH, DO, TUR and EC.
Elijah et al. [35]	2018	LoRaWAN, NB-IoT	pH, BOD, COD, AN, suspended solid (SS) and DO
Di Gennaro et al. [73]	2019	Sigfox	pH and TUR.
Chen and Han [61]	2018	Wi-Fi	DO, EC, TUR, pH and ORP.
Liu et al. [117]	2018	LoRaWAN	Temp, TUR, EC and pH.
Suryawanshi and Khandekar [118]	2018	Zigbee	pH and TUR.
Priya et al. [119]	2018	Zigbee/Wi-Fi	pH, EC, ORP, Temp and TUR.
Puneeth et al. [120]	2018	Wi-Fi	pH, TUR and Temp.
Das and Jain [121]	2017	Zigbee,GSM	pH, EC and Temp
Jindal et al. [54]	2017	Bluetooth/Wi-Fi	Temp, DO and EC
Kamaludin and Ismail [59]	2017	RFID	pH
Khaleeq et al. [122]	2017	Wi-Fi	pH, EC and Temp
Parameswari and Moses [62]	2018	Wi-Fi	Temp, pH, EC and TUR
Kafli and Isa [123]	2017	GSM	Temp, humidity, CO and pH
Salim et al. [124]	2017	Wi-Fi	Temp, pH, DO, TUR, EC, TDS and Salinity
Myint et al. [125]	2017	Zigbee	pH, TUR, CO2 and Temp
Cloete et al. [126]	2016	Zigbee	pH, EC, Temp and ORP
Raut and Shelke [127]	2016	Zigbee	pH, TUR
Ionel et al. [66]	2015	GPRS	Nitrate (NO^3−^), copper(Cu^++^), chloride(Cl^−^).
Wiranto et al. [128]	2015	GSM/GPRS	DO and pH
Adamo et al. [67]	2015	GSM/GPRS	Temp, EC, TUR and chlorophyll
Cook et al. [60]	2014	RFID	NOx
Rekhis et al. [56]	2012	RFID	pH, Temp, DO and TUR
Postolache et al. [57]	2011	RFID	Temp, pH, EC and TUR
Silva et al. [75]	2011	WiMax/Zigbee	DO, EC, Temp, pH and ORP

### 4.3 Machine learning applications on WQM

**Fig. 11:**
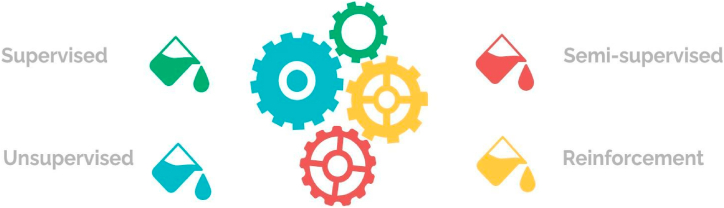
Different types of machine learning.

IoT technologies ensure WQ collection from various sources, including sensors, satellites, and citizen science initiatives. ML with its different techniques (Fig. 11) has emerged as a powerful tool for analyzing and predicting WQ parameters, enabling the identification of patterns, relationships, and anomalies that traditional statistical methods may not readily discern. By leveraging these insights, WQ managers can make more informed decisions about managing and protecting water resources, preventing pollution, and maintaining ecosystem health. In this section, we focus on supervised and unsupervised learning techniques inWQM. Supervised Learning uses labeled data sets to develop models that predict specific WQ parameters, while unsupervised learning identifies patterns and relationships in unlabeled data sets to inform decision-making. Understanding the applications of both techniques provides insight into how ML can improve water resource management practices.

#### 4.3.1 Supervised learning

ML is a natural outgrowth of the intersection of Computer Science and Statistics [129]. Supervised learning is a branch of Machine Learning. It uses labeled datasets to train algorithms capable of categorizing input and predicting outcomes. Its origins may be traced back to the 1950s and 1960s, when scientists developed linear regression models to investigate and predict correlations between variables. Supervised learning trains a model using previously labeled data or input/output pairs to predict future results. To reliably predict outputs from novel inputs, a function that may be reasonably approximated must be built. Two sorts of challenges that may occur with supervised learning are regression and classification [130]. Researchers began developing more complex supervised learning algorithms in the 1970s and 1980s, as computers became more widely available. For example, decision trees (DT) were developed in the 1970s as a tool to represent complex decision-making processes [131].

ANN have significantly advanced supervised learning by allowing the modeling of intricate nonlinear interactions between variables. Interest in supervised learning has been rekindled by the availability of big datasets and better computer capabilities. A variety of techniques, including support vector machines (SVM), random forests (RF), and DL algorithms, have been created and used by researchers in a variety of domains, including remote sensing [132], computer vision [133], image processing ([[134], [135]]) natural language processing [136], health [137], andWQM. Bagging, k-nearest Neighbors (k-NN), Logistic Regression (LR), Naive Bayes, and Bayesian Networks are more examples of supervised learning algorithms.

Supervised Learning models have been extensively studied for their application in WQM and prediction. Among these models, ANFIS algorithm has emerged as a particularly effective tool. Hmoud Al-Adhaileh and Waselallah Al- saade [27] focused on developing ANFIS to evaluate WQI. Additionally, they employed k-NN and feed-forward neural network (FFNN) techniques for WQ classification. Their research showcased the versatility of ANFIS in addressing different aspects of WQ analysis. The FFNN method had the greatest classification accuracy.

Another study conducted by Pham et al. [138] recognized the strength of ANFIS and utilized it alongside ANN and the group data processing approach (GMDH) to forecast WQI. The results clearly indicated that ANFIS outperformed other techniques employed in their comparison. This finding highlights the superior predictive capability of ANFIS in WQ forecasting tasks. Similarly, Abba et al. [139] proposed a comprehensive approach for WQI forecasting along the Yamuna River in India. Their methodology incorporated various models, including Backpropagation neural networks (BPNN), ANFIS, Support Vector Regression (SVR), and Multiple Linear Regression (MLR). By doing so, they aimed to harness the power of ANFIS in combination with other models to achieve accurate WQI predictions. In a study conducted at Bansong in South Korea, a hybrid approach combining ANFIS with k-means clustering was proposed. The findings reveal that the sub-models generated by the k-means-ANFIS hybrid outperformed both standalone ANFIS models and seasonal models constructed using ANN. By harnessing the power of ANFIS, this approach proved effective in accurately forecasting settling water TUR and determining the most suitable coagulant dosage [140].

In WQ research, there have been notable contributions focusing on the application of ANN. Imani et al. [141] aimed to enhance WQ resiliency, while Srivastava et al. [52] focused on forecasting WQI. Furthermore, Deng et al. [142] developed a model to predict algal growth by employing two ML techniques: ANN and SVM. For this model, water temperature, flow travel time, and DO were selected as initial inputs, enabling the estimation of total nitrogen (TN) and TP concentrations at any given point in the river. Interestingly [143], found that SVM models exhibited higher prediction accuracy compared to equivalent ANN models. Moreover, Guo et al. [144] investigated water-quality monitoring in small urban water bodies and evaluated three ML models: RF, SVR, and ANN.

RF algorithm has proven to be a valuable tool in forecasting various parameters for groundwater monitoring. In studies comparing different ML models, including Adaptive Boosting (AdaBoost), RF, and SVR. RF consistently exhibited superior performance. Researchers have successfully employed RF and Adaboost models to forecast parameters such as potential salinity, sodium adsorption ratio, and exchangeable sodium percentage, utilizing inputs such as EC, Temp, and pH [145].Similarly, researchers have explored the combination of RF with other algorithms to enhance prediction accuracy. For instance, Lu and Ma [146] developed Empirical Mode Decomposition with Adaptive Noise (CEEMDAN)-RF and CEEMDAN-XGBoost models by incorporating RF or eXtreme Gradient Boosting (XGBoost) algorithms with CEEMDAN. These models successfully predicted water temperature, DO, pH value, specific conductance, TUR, and fluorescent dissolved organic matter, showcasing the effectiveness of RF in capturing complex relationships within WQ data. Valerio et al. [26] utilized RF in conjunction with Gradient Boosted Regression Trees (GBRT) to monitor real-time WQ parameters and assess the impact of anthropogenic stressors on freshwater flora and fauna. This approach allowed them to gain insights into the intricate dynamics between WQ and ecological systems, with RF contributing to accurate predictions and a comprehensive understanding of the environmental factors at play.

Regarding specific parameters, Heddam and Kisi [147] estimated DO concentration using Extreme Learning Machines (ELM) models, outperforming Multi-Layer Perceptron Neural Network (MLPNN) and MLR. Chen et al. [148] used a convolutional Neural Network (CNN) architecture and DT algorithm to determine the COD values of water samples. Ewusi et al. [149] predicted TDS content in groundwater, surface water, and drinking water using hybrid predictive models combining Gaussian Process Regression (GPR), BPNN, and Principal Component Regression (PCR). Barzegar et al. [150] predicted EC using ELM and wavelet-extreme learning machine hybrid (WA-ELM) models, with the hybrid models exhibiting higher performance. Shah et al. [24] estimated both TDS and EC using Gene Expression Programming (GEP), ANN, MLR, and Multinomial Logistic Regression (MNLR) methods, with GEP showing the best performance. For the estimation of Escherichia coli (E.coli) levels, Wang et al. [151] employed linear regression models (MLR, PLS, and SPLS) and nonlinear models (RF and Bayesian Network) as input features. Li et al. [152] assessed E. coli levels and other indicators of effluent WQ, while Deng [142] used ANN and SVM to predict algal growth.

#### 4.3.2 Unsupervised learning

Unsupervised learning is a form of self-organization in which learning takes place without the involvement of a teacher. As researchers investigate its potential and importance in expanding our understanding of learning processes, it has emerged as a prevailing field in neural networks [153]. It was first proposed in the 1950s, but it only became widely accepted after the development of clustering techniques like k-means and hierarchical clustering (HC), as well as the application of neural networks [154]. The shift from supervised to unsupervised learning methods marks a significant change in how data exploration is approached, particularly in WQM. Supervised learning relies on labeled datasets to make precise predictions, while unsupervised learning delves into uncharted territories without explicit labels. This transition unlocks the potential of vast amounts of unlabeled data to unveil hidden patterns, anomalies, and latent structures. Unlike the explicit predictions of supervised learning, unsupervised methods identify hidden patterns, complex relationships, and emerging groupings within datasets. When combined supervised techniques allow for targeted forecasts, while unsupervised techniques reveal underlying trends, providing a more thorough grasp of the dynamics of WQ and a wider range of insights.

The emergence of DL and generative models has resulted in a greater emphasis on unsupervised learning use inWQM [155], which employs a variety of methods to detect patterns, connections, and structures in data that lacks labeled information. Applications include dimension reduction, cluster analysis, and anomaly detection. These techniques offer important new understandings of the origins of pollution, seasonal patterns, spatial and temporal variability [156], and the identification of newly emerging contaminants. These approaches can reveal previously unknown correlations between WQ parameters, allowing for a more in-depth knowledge of WQ dynamics and underlying mechanisms. Anomalies or deviations from the standard can also be detected, allowing for early warning systems and preventive maintenance. Unsupervised learning algorithms may also segment and categorize WQ data, decrease the dimensionality of high-dimensional data, identify latent variables and hidden elements affecting WQ trends [157]. Unsupervised learning techniques' potential to improve WQ assessment, monitoring, and management will grow as they evolve and integrate with IoT-driven WQM systems, paving the way for a more comprehensive and proactive approach to protecting water resources.

PCA played a pivotal role in many studies. Krishnaraj and Deka [156] utilized unsupervised ML techniques, including cluster analysis (CA) and PCA, to investigate the spatiotemporal pattern of river WQ in the Ganga River Basin. Notably, PCA was employed to establish correlations among variables such as EC, pH, TDS, T, Ca, Cl, HCO3, Mg, NO2+NO3, SiO2, and DO. Additionally, the researchers used PCA in conjunction with the K-means clustering method to group the monitoring stations into distinct clusters based on the dry and wet seasons. Likewise, Cao et al. [158] leveraged PCA to identify the main variables influencing fluctuations in DO. By applying PCA, they extracted the essential components associated with DO variations. Subsequently, DO time series data was organized, and predictions were made using both K-means clustering and a Gated Recurrent Unit (GRU) neural network. Haghnazar et al. [159] investigated the potential sources of heavy metals in groundwater and their relationships with hydro-chemical parameters. In their study conducted in the Urmia basin, known for hosting the largest lake in the Middle East, PCA was employed in combination with Pearson correlation analysis. This allowed for a comprehensive exploration of the associations between hydro-chemical parameters and the sources of heavy metals. PCA was a key component in the study conducted by Al-Sulttani et al. [160], where they integrated solo models such as Quantile Regression Forest (QRF), RF, SVM, and Gradient Boosting Machines (GBM) with different feature extraction methodologies. The performance evaluation of these models revealed that PCA-QRF model proposed in their study exhibited significantly better performance compared to both the standalone models and the Genetic Algorithm (GA)--integrated models, specifically about BOD. This underscores the crucial role played by PCA in improving the effectiveness and overall performance of the integrated model.

PCA proved to be an invaluable tool in these studies, facilitating the identification of crucial variables, establishing correlations, grouping data, and extracting essential components for further analysis. Its application enabled dimensionality reduction and provided valuable insights into the relationships and patterns within the datasets, enhancing the understanding of spatiotemporal variations and sources of WQ parameters and heavy metals. Numerous studies have utilized k-means to explore and analyze various datasets. For instance, Visalakshi and Radha [161] employed the k-means algorithm to divide a dataset into clusters, enabling the identification of outliers within each cluster through outlier detection techniques. This approach enhanced the understanding of the drinking water dataset and facilitated the identification of abnormal instances. In another study by Mohammadrezapour et al. [162], clustering techniques, including k-means, were applied to determine homogeneous zones based on groundwater quality (GWQ). By analyzing hydrochemical parameters from wells, the researchers identified distinct clusters using both k-means and Fuzzy C-means (FCM) algorithms. This clustering analysis helped in delineating different zones characterized by GWQ patterns. Moreover, Kim and Parnichkun [140] proposed a hybrid approach combining k-means with ANFIS. They applied this approach to forecast settling water TUR and optimize coagulant dosage at a Water Treatment Plant. The k-means-ANFIS hybrid outperformed standalone ANFIS models and seasonal models constructed using ANN, indicating the effectiveness of incorporating k-means clustering into the prediction framework.

By leveraging the power of k-means clustering, these studies have demonstrated its usefulness in various domains, including outlier detection, zoning analysis, and prediction modeling. The ability of k-means to partition data into clusters based on similarities has provided valuable insights, enabling researchers to uncover hidden patterns and structures in the datasets they investigated.

Groundwater plays a central role in many studies, which focuses on its pollution source identification and apportionment. Meng et al. [163] conducted an investigation to determine potential pollution sources and analyze their spatial distribution in groundwater between 2006 and 2016. They employed the Absolute Principal-Component-Score Multiple-Linear Regression (APCS-MLR) and PCA/Factor Analysis (FA) methodologies. The results highlighted that heavy metals (iron and manganese), nitrogen compounds (ammonia nitrogen, nitrite, and nitrate), and organic pollution (COD) were the primary contributors to groundwater pollution. Zhang et al. [164] focused on groundwater pollution source identification and apportionment. They utilized the Probabilistic Matrix Factorization (PMF) and PCA-Adaptive Principal Component Analysis (APCA)-MLR receptor models for their analysis. This integrated approach, incorporating PCA, aimed to accurately identify and allocate pollution sources in groundwater, providing valuable insights for effective environmental management in the region.

**Fig. 12:**
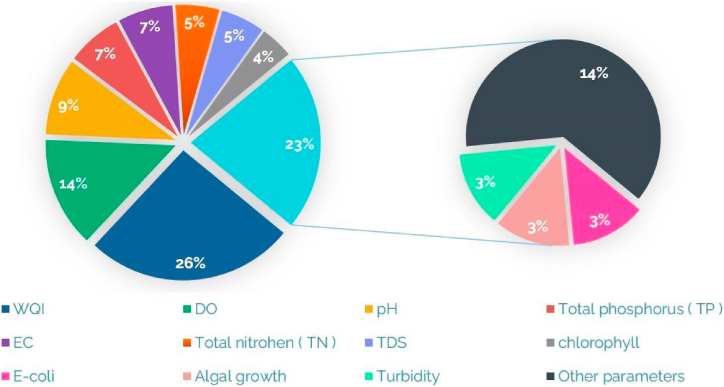
Predicted pollutants: Percentage distribution in WQM

**Fig. 13:**
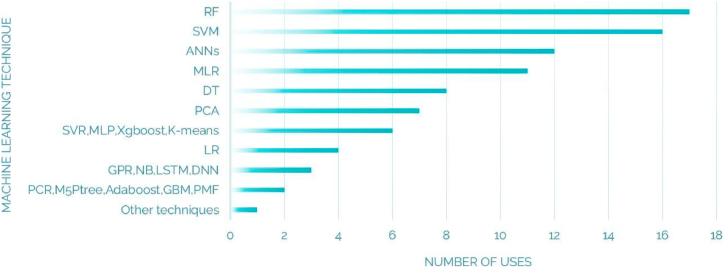
Frequency of machine learning methods used in WQM

Table 2 provides a comprehensive overview of the ML techniques utilized in the review. Fig. 12 visually represents the percentage distribution of predicted pollutants in the review, giving us a clear understanding of how prevalent and significant each contaminant is. Additionally, Fig. 13 shows the frequency of ML methods used, giving us an idea of the popularity and effectiveness of different algorithms in analyzing WQ data. These figures contribute to a better understanding of the research findings, providing both visual and quantitative information about the ML techniques and their impact on WQ assessment.

**Table 2:** Machine Learning techniques for WQM: A table Overview.

Reference	Year	ML techniques	Purpose
Jamshid Zadeh et al. [165]	2024	BiLSTM, SVM	EC, TDS
Rahu, Mushtaque Ahmed et al. [77]	2024	SVM, RF, linear regression, Naive Bayes, and DT	Forecasting Agricultural Water Needs
Uddin et al. [166]	2023	GPR	Predicting WQI
Hu et al. [167]	2023	Least Absolute Shrinkage and Selection Operator (Lasso), PCR, Resilient Backpropagation (RPROP), Generalized Regression Neural Network (GRNN), Bidirectional Recurrent Neural Network (BRNN), RF, SVR, GPR,MLR	Forecasting the formation of disinfection byproducts (DBPs).
Omeka [168]	2023	MLP-ANNs, MLR	Predicting WQI
Uddin et al. [169]	2023	SVM, Naïve Bayes (NB), RF, k-NN, XG- Boost	Predicting WQI
Lap et al. [170]	2023	LR, MLP, SVM, DT, RF	Predicting WQI
Yan et al. [171]	2023	SVM, RF, Adaboost, and gradient boosting decision tree (GBDT), the Bayesian algorithm	Predicting WQ levels
Narita et al. [172]	2023	RF, XGBoost, and LightGBM,	Forecasting pesticide detectability in surface water
Chen et al. [173]	2023	RF, GBRT, XGBoost, Deep Neural Network (DNN)	Predicting chlorophyll (Chla), TUR, ammonia nitrogen
Boccadoro e tal [74].	2022	Long-Short-Term-Memory (LSTM)	Predicting pH, EC, DO, and Temp,
Nasir et al. [174]	2022	SVM, RF, LR, DT, XGBoost, MLP, Categorical Boosting (CATBoost),	Predicting WQI
Li et al. (2022) [85]	2022	K-means, PCA	Selection of WQ key parameters
Azrour et al. [175]	2022	Regression algorithms	Predicting WQI
Uddin et al. ([176]	2022	RF, DT, k-NN, XGBoost, Extra Tree, (ExT), SVM, LR, and Gaussian Naïve Bayes (GNB)	Predicting WQI
Khullar and Singh [177]	2022	Bidirectional LSTM(Bi-LSTM)	Predicting COD and BOD.
Nourani et al. [178]	2022	K-Means, agglomerative hierarchical method of Ward, Growing Neural Gas (GNG)	Comprehending changes in groundwater quantity and quality
Zai et al. [179]	2022	DT	Predicting WQI
Kadkhodazadeh and Farzin [180]	2022	least-squares support vector machine- arithmetic optimization algorithm (LSSVM-AOA)	Estimating TDS and EC.
Haghnazar et al. [159]	2022	PCA-MLR	Evaluating GWQ
Kokatnoor et al. [181]	2022	BRR, automatic relevance determination regression (ARD)	Predicting WQI
Gómez et al. [182]	2021	RF, SVM, ANN, DNN	Estimating chlorophyll-a (chl-a)
Tousi et al. [183]	2021	SVM, LR, ridge classifier (RC)	Classifying E. coli
Kouadri et al. [184]	2021	MLR, RF, M5P tree (M5P), random subspace (RSS), additive regression (AR), ANN, SVR, and locally weighted linear regression (LWLR)	Predicting WQI
Ewusi et al. [149]	2021	GPR, BPNN, PCR	Predicting TDS
Deng et al. [142]	2021	ANN, SVM	Predicting algal growth and eutrophication
Imani et al. [141]	2021	ANN	Predicting WQI
Al-Sulttani et al. [160]	2021	QRF, RF, SVM, GBM, GA, PCA	Predicting BOD.
Wang et al. [151]	2021	MLR, PLS, SPLS, RF, BN	Predicting E.coli
Shah et al. [24]	2021	GEP, ANN, MLR, MNLR	Predicting TDS, EC
Valerio et al. [26]	2021	RF, GBRT	Predicting the influence of human-induced stress factors on freshwater ecosystems
El Bilali et al. [145]	2021	Adaboost, RF, ANN, SVR	GWQ forecasting: TDS, PS, SAR, ESP, MAR and RSC
Guo et al. [144]	2021	RF, SVR, ANNs.	Predicting: TP, TN, and COD
Lu and Ma [146]	2020	XGBoost, RF, CEEMDAN	Predicting Temp, DO, pH, TUR, conductance, and fluorescent DOM
Li et al. [152]	2020	normal equation linear regression, stochastic gradient descent (SGD), and Ridge regression (RR)	Predicting nitrogen, phosphorus, total coliform, E. Coli, TDS
Xu et al. [185]	2020	k-NN, GBDT, SVM, MLP	Predicting Fecal Indicator Bacteria (FIB)
Bui et al. [186]	2020	RF, M5P, random tree (RT), and reduced error pruning tree (REPT)) and 12 hybrid data-mining algorithms	Predicting WQI
Chen et al. [148]	2020	CNN, DT	Predicting COD
Krishnaraj and Deka [156]	2020	CA, PCA	Understanding spatial and temporal trends in river water quality
Cao et al. [158]	2020	PCA, K-means, GRU	Predicting DO
Zhang et al. [164]	2020	PMF, PCA, APCA, MLR	Identifying the sources of contamination in groundwater.
Radhakrishnan and Pillai [187]	2020	SVM, DT, NB	Predicting pH, TUR, EC, DO, nitrate, Temp and BOD
Jalal and Ezzedine [188]	2020	SVM, DT	Anomaly Detection in Water
Abba et al. [139]	2020	BPNN, ANFIS, SVR, MLR	Predicting WQI
Mohammadrezapour et al. [162]	2020	KM, FCM, GA	Identification of homogeneous regions of GWQ
Muharemi et al. [189]	2020	SVM, ANN, DNN, recurrent neural net-work (RNN), LSTM	Anomaly Detection in Water
Najah Ahmed et al. [190]	2019	ANFIS, Radial Basis Function Neural Networks (RBF-ANN), MLP-ANN	Predicting WQI
Budiarti et al. [113]	2019	SVM	Predicting Pollution index
Abbas et al. [23]	2019	ANN, DT	Predicting WQI
Meng et al. (163]	2019	APCS-MLR, PCA, factor analysis (FA)	Comprehending alterations in the quality of groundwater.
Cao et al. [191]	2018	Least Squares SVR (LSSVR)	Predicting pH, DO, COD and NH3
Chen et al. [192]	2018	SVM	Using water imagery for assessing WQ.
Dezfooli et al. [193]	2018	probabilistic neural network (PNN), k-NN, SVM	Categorizing WQ through parameters such as TUR, fecal coliform levels, and total solids content
Barzegar et al. [150]	2018	WA-ELM, WA-ANFIS	Predicting EC
Srivastava et al. [52]	2018	ANN	Predicting WQI
Heddam and Kisi [147]	2017	MLPNN, MLR, ELM	Predicting DO
Kim and Parnichkun [140]	2017	k-means-ANFIS	Predicting TUR
Wang et al. [29]	2017	LSTM, NN	Predicting DO and TP
Visalakshi and Radha [161]	2015	K-means	Enhancing the identification of anomalies in extensive, multi-dimensional datasets.
Sharif et al. [194]	2015	K-means, Self-Organizing Map (SOM)	Analyzing the spatial and temporal trends in WQ data while pinpointing pollution sources.
Liu and Lu [143]	2014	ANN, SVM	Predicting TN and TP
Liu Fu-cheng and He Xue-zhao [195]	2013	FCM	Categorizing and evaluating the quality of rural surface water using WQI.

## 5 Discussions

### 5.1 Findings and challenges

In the quest to efficient WQM, the use of IoT and ML technologies has become increasingly popular for WQM. In this survey article, we delved into the different networks commonly used in WQM, as well as the most frequently monitored parameters.

In terms of parameters, our survey article found that pH, Temp, TUR, EC, DO, humidity, and TDS are the most frequently monitored parameters for WQ in the context of IoT-based systems. These parameters are important for assessing the health of aquatic ecosystems, detecting contamination events, and supporting regulatory compliance. Monitoring these parameters can enable real-time detection of WQ changes, making it possible to respond quickly to potential contamination events and improveWQM.

The frequency of WQ parameter monitoring is influenced by practical considerations and cost, as well as the specific concerns related to the WQ at the monitored site and the objectives of the monitoring program. Fortunately, the measurement of these parameters is straightforward, thanks to the accessibility and affordability of sensors. An effective strategy in WQM programs involves tracking variations in pH and Temp, which can serve as early indicators of potential WQ issues. However, the inherent unpredictability of factors like temperature and pH can make it challenging to detect changes caused by pollution. Therefore, it is crucial to account for this natural variability when analyzing WQ data and identifying long-term trends.

In contrast, some parameters may be less frequently monitored due to factors such as the cost and availability of sensors or the less immediate impact on WQ. For example, the monitoring of metals and metalloids can be expensive, and sensors for these parameters may not be as widely available as those for other parameters. Similarly, monitoring for nitrate and phosphate may be more important in agricultural areas where runoff can be a concern, but may be less relevant in other contexts. Emerging contaminants, such as microplastics, pharmaceuticals, and personal care products, are a growing concern in WQM. These contaminants are often present at low concentrations, and their effects on human health and the environment are not well understood. This makes it challenging to assess the risk they pose and identify appropriate monitoring strategies.

IoT-based WQM systems have numerous advantages over traditional methods such as Real-time WQM. Because of the improved data gathering and analysis made possible by this real-time monitoring, the outcomes are more precise. Additionally, because of their high scalability and affordability, these systems are ideally suited for low-income communities and developing nations. In such areas, where WQM is crucial but may be financially challenging, IoT-based systems provide a viable solution.

Nevertheless, current monitoring practices have their limitations. These practices only occur in specific locations and monitor limited parameters, which may not provide a comprehensive understanding of WQ and potential contaminants. Additionally, the frequency of monitoring could miss transient changes that have a big effect on the environment and human health. The high expense of monitoring can also be a barrier, especially in low-income areas and developing nations where it may be difficult to construct monitoring systems.

Low-quality sensors in WQM can lead to incomplete or inaccurate data, resulting in incorrect assessments of WQ. In addition to being less reliable and requiring more frequent calibration or replacement, low-quality sensors can lead to false alarms or missed detections, hindering the development of effective management strategies to mitigate water pollution and ensure the safety of water resources.

One of the major challenges in IoT-based WQM is selecting the appropriate sampling frequency and location. This is because the ideal frequency and location vary based on the water body being monitored, the location, and the pollutants present. Additionally, the sensitivity of analytical methods used in WQM is a limitation, with some contaminants being present at very low concentrations that standard methods may not detect. This requires more advanced and expensive equipment. The lack of standardization across regions or jurisdictions further complicates data comparison and the development of a comprehensive national or global picture of WQ.

Our research shows that LPWAN and cellular networks have become widely utilized in water monitoring applications. These networks are widely available, and the necessary infrastructure is already in place in many locations, making them a convenient and cost-effective option for deploying IoT devices and sensors. However, both LPWAN and cellular networks encounter challenges related to coverage gaps, which can hinder their effectiveness in ensuring ubiquitous connectivity. LPWAN technologies like LoRaWAN and Sigfox offer long-range communication with low power consumption, but they face coverage gaps due to limited infrastructure deployment and difficulties in penetrating dense urban areas or remote regions. Cellular networks, encompassing various generations like 2G, 3G, and 4G LTE, have notably enhanced connectivity by offering higher data rates and broader coverage. However, despite these advancements, coverage gaps endure in specific geographical areas, notably in rural regions or underground environments(Zhang et al., 2021) [196]. The reliance of WQM systems on LPWAN and cellular networks for consistent data transmission renders them highly susceptible to severe disruptions caused by these network coverage gaps. These breaches cause data loss or disappearance from sensors situated in these places, which obscures critical information regarding WQ indicators and possible pollution occurrences and leaves monitoring gaps. This leads to a lack of environmental oversight, which affects the system's capacity to fully monitor circumstances. Furthermore, data transmission delays brought on by sporadic coverage lessen the system's ability to react quickly to critical events like abrupt spikes in contamination. The reliability of the system is further compromised by coverage gaps that cause communication disruptions between sensors and the central monitoring platform. These disruptions may result in issues with sensor management, design and coverage problems [197]. The advent of 5G technology brings substantial advancements in wireless communications, with higher data rates, lower latency, and improved spectral efficiency, making it capable of addressing coverage gaps in LPWAN and cellular networks.

This survey article has found that LoRa is the most commonly used LpWAN technology for WQM. LoRaWAN is a wireless communication protocol that has gained popularity in the IoT industry because of its ability to transmit data over long distances with low power consumption. One of the main reasons why LoRaWAN is preferred over other LpWAN technologies, such as Sigfox and NB-IoT, is its longer range, which makes it well-suited for WQM applications that require long-range communication. LoRa's lower power consumption is also an advantage for WQM applications where sensors may need to operate for long periods on battery power. LoRaWAN is an open standard technology with a large community of developers and users who are actively working on developing new solutions and improving the technology. Finally, LoRaWAN is relatively cost-effective compared to other LpWAN technologies, as it does not require expensive infrastructure or licenses. This makes LoRaWAN an attractive option for implementing WQM solutions.

When it comes to WQM, LoRaWAN is commonly used as a LpWAN technology. However, there are instances where other LpWAN technologies like NB-IoT and Sigfox might be more suitable. NB-IoT has the advantage of operating within existing cellular networks, making it easier to deploy in urban areas where there is already cellular coverage. This can be beneficial for WQM in densely populated areas with a higher concentration of potential contamination sources. On the other hand, Sigfox is optimized for transmitting small amounts of data over long distances while consuming minimal power. This makes it well-suited for WQM in remote locations where there is no cellular or internet connectivity available, such as rural or isolated areas. Ultimately, the choice of LpWAN technology depends on the specific requirements of the application, including the desired range, data rate, power consumption, and the availability of infrastructure.

Wi-Fi technology is preferred for WQM because of its practicality, affordability, real-time monitoring capabilities, and data management features. There are restrictions on its use, nevertheless, that must be taken into account. Wi-Fi signals have a limited range, making them less appropriate for monitoring in rural regions or vast bodies of water that are not connected to a Wi-Fi network. The accuracy of data gathered by Wi-Fi-equipped sensors can potentially be impacted by interference from other electrical devices. Wi-Fi sensors also need a power source, which might be difficult to provide in rural locations without access to energy. security vulnerabilities in Wi-Fi networks could jeopardize the accuracy of data gathered by Wi-Fi-equipped sensors.

The findings of the state-of-the-art paper suggest that RFID, Bluetooth, and Zigbee technologies are less commonly used in WQM compared to LpWAN and cellular networks. Nonetheless, Zigbee, RFID, and Bluetooth are preferred in low-power, short-range, and low-cost communication situations where a large number of small devices need to communicate with each other in a confined area. Zigbee can create a mesh network of sensors, which can transmit data to a nearby gateway, a cost-effective and power-efficient alternative to cellular networks that consume more power and require dedicated network infrastructure. RFID and Bluetooth are beneficial for localized data collection scenarios in WQM.

But even with these advances, problems remain, particularly with regard to signal interference. Within residential environments, problems with transmission attenuation and interference from reflecting surfaces arise for communication protocols like Wi-Fi, Bluetooth, and ZigBee. Variations in the power transmission ranges of wireless devices within residences might lead to possible issues with signal interference. Maintaining dependable connection across several wireless devices requires effective interference management [198]. When it comes to sensor deployment, careful thinking and analysis are required to address the interference risk. It is crucial to comprehend the intricate details of signal overlap and investigate methods to reduce interference-induced false alarms.

Over half (51%) of industry professionals believe that Industrial Control Systems (ICS) do not have enough protection, according to a 2020 analysis based on data from the USA. Furthermore, 55% of respondents are concerned about these systems' susceptibility to cyberattacks [199].

The water and wastewater sector ranks as the third vulnerable sector susceptible to cyberattacks [200]. Which constitutes a significant concern, especially considering the integration of SCADA (Supervisory Control and Data Acquisition) systems in monitoring and controlling essential infrastructure like water distribution networks. SCADA systems, which are a type of Industrial Control System (ICS), oversee large geographic areas and are pivotal in managing vital utilities such as gas pipelines, electric power transmission, and water distribution systems. the vulnerabilities of SCADA systems, can endanger public health and the environment. Hackers can exploit these vulnerabilities to manipulate water treatment processes, increase chlorine concentration beyond safe levels, disrupt transportation tanker tracking systems, and cause environmental disasters. To mitigate these risks, industry stakeholders and government authorities should implement robust cybersecurity measures, foster collaboration among stakeholders, and strengthen the security posture and resilience of critical infrastructure systems [201].

Water purification and distribution systems need a multi-layered security approach to protect against cyber threats. This includes regular vulnerability assessments, robust security measures across physical, cyber, and network domains, and safeguarding sensitive data. Fault tolerance mechanisms ensure uninterrupted operation, and advanced attack detection techniques like ML aid in early detection and mitigation. Standardized interfaces and protocols enhance interoperability, and employee training in cybersecurity best practices prevents social engineering attacks. Intrusion detection and prevention systems monitor malicious activities, while data encryption secures sensitive information. Multi-factor authentication adds an extra layer of security. Continuous monitoring, resilience testing, collaboration, and ongoing research are essential for a robust security posture) [200].

Based on The survey of the use of ML techniques for WQ analysis, the most predicted parameters are WQI, (DO, pH, EC, and TP. These parameters are widely employed in the analysis of WQ, and their accurate prediction is crucial for effective monitoring and management of water resources. Leveraging ML techniques for predicting these parameters can offer valuable insights into trends, aid in identifying potential pollution sources, and facilitate decision-making in WQM. However, it is important to recognize that relying solely on these parameters may oversimplify the assessment of WQ, potentially overlooking its intricate complexities and resulting in insufficient or misleading evaluations. Furthermore, the absence of standardized practices in parameter usage can impact the comparability of results across various studies. This approach may not consider the interactions between parameters, which can lead to incomplete or incorrect assessments of WQ. This highlights the need to consider a broader range of parameters and to standardize their use to ensure comprehensive and comparable assessments of WQ. Additionally, to give reliable assessments of WQ, it is also crucial to take into account regional and temporal fluctuations as well as the interactions between parameters.

Based on the survey paper, the most commonly used algorithms in WQM are designed for regression or classification purposes, as well as clustering and dimensionality reduction. Among the most commonly used algorithms are random RF, SVM, ANNs, MLR, DT, PCA, k-means clustering, SVR, MLP, Xgboost, and LR.

In WQM, algorithm selection relies on factors such as monitoring program goals, data type, volume, and available resources for analysis. The choice of algorithm is influenced by the strengths and limitations of each. For instance, some algorithms such as RF and SVM are better suited for classification tasks, while ANNs are useful for modeling complex relationships between predictors and outcomes. MLR is often used to model the relationship between environmental variables and WQ parameters, while DT is effective in identifying the most significant predictors. PCA is useful in detecting patterns in data, and k-means clustering can identify groups of samples with similar WQ characteristics.

The selection of algorithms should be carefully considered, taking into account the specific research question and the characteristics of the data under analysis. Regression algorithms are valuable tools for predicting WQ parameters like pH, DO, and nutrient concentrations. However, they might struggle to capture complex relationships between these parameters and environmental factors such as temperature or land use. For handling non-linear relationships, tree-based algorithms prove to be more suitable, although their accuracy can be compromised when dealing with high-dimensional data. On the other hand, algorithms like SVR, multilayer perceptron (MLP), and Xgboost are effective options for modeling complex non-linear relationships and handling large and intricate datasets with numerous predictors. SVR and MLP, in particular, exhibit prowess in capturing intricate nonlinearities. Xgboost, on the other hand, shines when it comes to managing complex datasets with a multitude of predictors. Another commonly utilized algorithm in WQM is LR. LR is frequently employed to predict the probability of a binary outcome variable based on one or more predictor variables. It is also adept at estimating the likelihood of surpassing regulatory thresholds, making it a valuable tool in regulatory compliance assessments. Clustering algorithms offer valuable assistance in identifying similar WQ samples, aiding in the exploration of patterns and similarities among different observations. However, the accuracy of clustering results can be subjective and dependent on the user's interpretation. For visualizing high-dimensional WQ data and identifying important features, dimensionality reduction techniques come into play. These techniques help simplify the data and enable easier visualization. Nevertheless, it is crucial to note that in the process of dimensionality reduction, relevant information may be discarded, potentially oversimplifying the data.

### 5.2 Future prospects and emerging trends in WQM

In recent years, the water sector has undergone a remarkable transformation, spurred by advancements in data science, analytics, and digital technologies. These advancements have revolutionized WQM practices, with ML algorithms and techniques emerging as a pivotal force in shaping this future. ML plays a crucial role in data analytics, empowering water utilities to extract valuable insights from complex datasets and make informed decisions. By continuously analyzing real-time data collected through sensors and IoT devices, machine learning models enable real-time assessment of WQ parameters, anomaly detection, and optimization of water treatment processes. The integration of ML into intelligent water networks has significantly enhanced water distribution efficiency. ML algorithms can detect leaks, optimize pressure levels, and improve overall system performance, ensuring a reliable and sustainable water supply. ML also holds immense potential for promoting water conservation. By analyzing smart meter data, ML techniques can identify abnormal consumption patterns, pinpoint leaks, and provide personalized insights to consumers. This empowers individuals to make informed choices and optimize their water usage. Predictive maintenance, driven by ML algorithms, has revolutionized maintenance practices by predicting equipment failures and maintenance requirements. This proactive approach reduces downtime, extends equipment lifespan, and optimizes resource allocation. Real-time WQM, facilitated by ML, has become a critical component of WQM. By detecting contamination events early, ML enables swift intervention and prevents the spread of waterborne diseases. Decision support systems integrated with ML models are transforming WQM by providing WQ professionals with real-time data and advanced analytics. This empowers them to make informed decisions regarding treatment strategies, resource allocation, and risk assessment, ensuring the timely and effective WQM challenges. These emerging trends in WQM, powered by ML, hold immense promise for ensuring the availability of clean and safe water for future generations. Machine learning is not only transforming WQM practices but also paving the way for a sustainable and resilient water future [202].

The future of WQM is intertwined with technological advancements and strategic innovations. IoT and ML technologies are poised to play an increasingly pivotal role in shaping this landscape. The quest for accurate and comprehensive WQ assessments necessitates the adoption of sophisticated sensors with an expanded detection range. These sensors, equipped with enhanced sensitivity and selectivity, can effectively capture the presence and concentration of various contaminants, enabling a more thorough evaluation of WQ parameters. The significance of high-quality resonators transcends the realm of WQ assessments, extending to various domains, including lasers, sensors, and optics. These resonators, capable of confining and amplifying electromagnetic waves, play a pivotal role in shaping light's behavior and enabling various applications. Fano resonance, a remarkable phenomenon, emerges from the interplay of distinct resonances within a material. This interplay produces unique spectral signatures that serve as distinctive fingerprints of the material's properties. Fano resonance not only enhances the resonances in the material but also introduces a sharp dip in the spectral response, a characteristic feature of this phenomenon. It is similar to electromagnetically induced transparency (EIT), an optical transparency phenomenon [203].

In sensing technologies, Fano resonances with high Q factors are crucial for improving sensor performance. A higher Q factor signifies a sharper and more distinct spectral response in the sensing system. This enhanced sharpness enables sensors to detect smaller changes in the environment or target parameters, improving sensitivity. Additionally, the narrower spectral response enhances selectivity, allowing sensors to discern specific targets or analytes from complex backgrounds more accurately [204].

These sensors can more precisely identify specific pollutants, decreasing false positives and enabling for early identification of contaminants. Bioreceptors and nanomaterials integrated into sensors will allow for the exact identification of new contaminants such as microplastics and medicines. However, the adoption of advanced technologies for WQM may be impeded by complex, expensive, and data management system problems. Standardization efforts must provide data comparability between research and geographic locations. Adoption is hampered by a number of factors, including a lack of expertise, expense, incompatibility with existing infrastructure, security, and privacy concerns, along with resistance to change, a lack of necessary skills, and inconsistent regulations, pose challenges to the adoption of large-scale IoT. The dynamic environment further complicates matters, while issues like evolving stakeholder roles and restricted co-creation stem from advanced technology [205]. To surmount these challenges, organizations have to develop communication, implement robust cybersecurity protocols, broaden their outreach and education initiatives, explore other funding options, and incorporate stakeholders in the decision-making procedures. Alternative finance models including grants, subsidies, and public-private partnerships can be used to promote adoption. Strong cybersecurity, data transparency, and infrastructure compatibility can all be increased through pilot projects and phased rollouts.

## 6 Conclusion

The utilization of ML and IoT in managing WQ has proven to be highly effective, revolutionizing the precision, speed, and efficacy of monitoring, analyzing, and predicting WQ. Consequently, smart water systems have been developed to instantly detect and address any fluctuations in WQ, ensuring the safety and security of water consumed, used for agriculture, and maintained for the environment.

The implementation of IoT wireless technologies has been crucial in advancing smart water systems for WQM. These technologies enable the gathering and analysis of vast amounts of data on WQ, facilitating the creation of accurate and reliable models for forecasting WQ. Real-time monitoring and response to changes in WQ, made possible by wireless sensors, communication networks, and cloud computing platforms, guarantee water safety for human consumption, agriculture, and the environment.

The application of ML algorithms in WQM has enhanced the efficiency of WQM, analysis, and prediction. Supervised ML algorithms have proven effective in predicting and classifying WQ parameters such as pH, Temp, TUR, and DO. These algorithms enable the identification of patterns and relationships within the data, leading to improved understanding and WQM. Unsupervised ML algorithms like clustering and anomaly detection have also been employed in WQM, enabling the detection and response to changes in WQ that may not be immediately apparent, allowing for corrective action before situations worsen. However, several challenges remain to be addressed. Standardized protocols for data collection, processing, and analysis are needed to ensure consistency in data quality. Additionally, the development of efficient and cost-effective IoT devices capable of withstanding harsh environmental conditions and maintaining accuracy and reliability over time is crucial.

Continuous monitoring and maintenance of IoT devices pose another challenge that must be tackled to ensure the longevity and effectiveness of WQM systems. Regular calibration of sensors, replacement of worn-out components, and software updates are essential maintenance practices for optimal functioning of IoT devices. Collaboration among researchers, policymakers, and water management authorities is essential in developing robust and sustainable solutions for WQM. Their collective efforts can result in effective and practical solutions to address the challenges faced in the application of ML and IoT in WQM. In conclusion, this survey paper highlights the significant contributions of ML techniques and IoT to the field of WQM. The successful integration of these advanced technologies has showcased their potential to accurately predict WQ parameters and promptly detect contaminants, promoting public health and environmental sustainability. Nevertheless, there are still areas for future research in this domain. Investigating the development of ML models capable of effectively handling missing or incomplete data, a common challenge in WQM, holds promise. Additionally, incorporating emerging technologies like blockchain and edge computing enhances WQM system precision and efficiency. Research efforts can also focus on optimizing sensor networks to improve reliability and reduce maintenance costs. Overall, the future of WQM appears promising, and ongoing research endeavors in this field will undoubtedly yield more effective and efficient systems for safeguarding our water resources.

## Funding

This review has received no funding

## Ethics

Not applicable.

## Data availability statement

No data was used for the research described in the article.

## CRediT authorship contribution statement

**Ismail Essamlali:** Writing – review & editing, Writing – original draft. **Hasna Nhaila:** Writing – review & editing. **Mohamed El Khaili:** Writing – review & editing, Writing – original draft.

## Declaration of competing interest

The authors declare that they have no known competing financial interests or personal relationships that could have appeared to influence the work reported in this paper.

## References

[1] Mekonnen M.M., Hoekstra A.Y. (2016). Four billion people facing severe water scarcity. Sci. Adv..

[2] Weststrate J., Dijkstra G., Eshuis J., Gianoli A., Rusca M. (2019). The sustainable development goal on water and sanitation: learning from the millennium development goals. Soc. Indicat. Res..

[3] Duran-Encalada J., Paucar-Caceres A., Bandala E., Wright G. (2017). The impact of global climate change on water quantity and quality: a system dynamics approach to the US–Mexican transborder region. Eur. J. Oper. Res..

[4] Allam Z., Dhunny Z.A. (2019). On big data, artificial intelligence and smart cities. Cities.

[5] Sjerps R.M., ter Laak T.L., Zwolsman G.J. (2017). Science of the Total Environment 601-602.

[6] Ritchie H., Spooner F., Roser M. (2023).

[7] Parisot A., Bento L.M., Machado R.C. (2021). 2021 IEEE International Workshop on Metrology for Industry 4.0 & IoT.

[8] Jiang J., Tang S., Han D., Fu G., Solomatine D., Zheng Y. (2020).

[9] Behmel S., Damour M., Ludwig R., Rodriguez M. (2016). Water quality monitoring strategies — a review and future perspectives. Sci. Total Environ..

[10] Karydis M., Kitsiou D. (2013). Marine water quality monitoring: a review. Mar. Pollut. Bull..

[11] Loaiciga H.A., Charbeneau R.J., Everett L.G., Fogg G.E., Hobbs B.F., Rouhani S. (1992). Review of ground-water quality monitoring network design. J. Hydraul. Eng..

[12] Prapti D.R., Mohamed Shariff A.R., Che Man H., Ramli N.M., Perumal T., Shariff M. (2022). Internet of Things (IoT)-based aquaculture: an overview of IoT application on water quality monitoring. Rev. Aquacult..

[13] Banna M.H., Imran S., Francisque A., Najjaran H., Sadiq R., Rodriguez M., Hoorfar M. (2014). Online drinking water quality monitoring: review on available and emerging technologies. Crit. Rev. Environ. Sci. Technol..

[14] Singh M., Ahmed S. (2021). Iot based smart water management systems: a systematic review. Mater. Today: Proc..

[15] Sagan V., Peterson K.T., Maimaitijiang M., Sidike P., Sloan J., Greeling B.A., Maalouf S., Adams C. (2020). Monitoring inland water quality using remote sensing: potential and limitations of spectral indices, bio-optical simulations, machine learning, and cloud computing. Earth Sci. Rev..

[16] Zhu M., Wang J., Yang X., Zhang Y., Zhang L., Ren H., Wu B., Ye L. (2022). A review of the application of machine learning in water quality evaluation. Eco-Environment & Health.

[17] Dogo E.M., Nwulu N.I., Twala B., Aigbavboa C. (2019). A survey of machine learning methods applied to anomaly detection on drinking-water quality data. Urban Water J..

[18] Ighalo J.O., Adeniyi A.G., Marques G. (2021). Artificial intelligence for surface water quality monitoring and assessment: a systematic literature analysis. Modeling Earth Systems and Environment.

[19] Ewuzie U., Bolade O.P., Egbedina A.O., Marques G., Ighalo J.O. (2022). Current Trends and Advances in Computer-Aided Intelligent Environmental Data Engineering.

[20] Wagle N., Acharya T.D., Lee D.H. (2020). Comprehensive review on application of machine learning algorithms for water quality parameter estimation using remote sensing data. Sensor. Mater..

[21] Page Matthew J. (2021). Updating guidance for reporting systematic reviews: development of the PRISMA 2020 statement. J. Clin. Epidemiol..

[22] Jerom B.A., Manimegalai R., Manimegalai R. (2020). 2020 International Conference on Emerging Trends in Information Technology and Engineering (Ic-ETITE).

[23] Abbas T., Mkelif A.M., Abdulkareem A.K. (2019). First International Conference of Computer and Applied Sciences (CAS).

[24] Shah M.I., Javed M.F., Abunama T. (2021). Proposed formulation of surface water quality and modelling using gene expression, machine learning, and regression techniques. Environ. Sci. Pollut. Control Ser..

[25] Islam M.M., Arefin M.S., Khatun S., Mokarrama M.J., Mahi A.M., Chen J.I.Z., Tavares J.M.R.S., Shakya S., Iliyasu A.M. (2021).

[26] Valerio C., De Stefano L., Martínez-Muñoz G., Garrido A. (2021).

[27] Hmoud Al-Adhaileh M., Waselallah Alsaade F. (2021). Modelling and prediction of water quality by using artificial intelligence. Sustainability.

[28] Edition F. (2011). Guidelines for drinking-water quality. WHO Chron..

[29] Wang Y., Zhou J., Chen K., Wang Y., Liu L. (2017). 2017 12th International Conference on Intelligent Systems and Knowledge Engineering (ISKE).

[30] Yan H., Liu Y., Han X., Shi Y. (2017). 2017 16th International Conference on Optical Communications and Networks (ICOCN).

[31] Che H., Liu S., Smith K. (2015). Performance evaluation for a contamination detection method using multiple water quality sensors in an early warning system. Water.

[32] Su X., Sutarlie L., Loh X.J. (2020).

[33] Horton R.K. (1965). An index number system for rating water quality. J. Water Pollut. Control Fed..

[34] Sutadian A.D., Muttil N., Yilmaz A.G., Perera B. (2016). Development of river water quality indices—a review. Environ. Monit. Assess..

[35] Elijah O., Rahman T.A., Yeen H.C., Leow C.Y., Sarijari M.A., Aris A., Salleh J., Han C.T. (2018). 2018 2nd International Conference on Smart Sensors and Application (ICSSA).

[36] Skarbøvik E., Roseth R. (2015). Use of sensor data for turbidity, pH and conductivity as an alternative to conventional water quality monitoring in four Norwegian case studies. Acta Agric. Scand. Sect. B Soil Plant Sci.

[37] Korostynska O., Mason A., Al-Shamma’a A.I., Mukhopadhyay S.C., Mason A. (2013).

[38] Lyons K.J., Ikonen J., Hokajärvi A.M., Räsänen T., Pitkänen T., Kauppinen A., Kujala K., Rossi P.M., Miettinen I.T. (2023).

[39] Gholizadeh M., Melesse A., Reddi L. (2016). A comprehensive review on water quality parameters estimation using remote sensing techniques. Sensors.

[40] Ahmed U., Mumtaz R., Anwar H., Mumtaz S., Qamar A.M. (2020). Water quality monitoring: from conventional to emerging technologies. Water Supply.

[41] Liu S., Che H., Smith K., Chen C. (2015). A method of detecting contamination events using multiple conventional water quality sensors. Environ. Monit. Assess..

[42] Martínez R., Vela N., El Aatik A., Murray E., Roche P., Navarro J.M. (2020). On the use of an IoT integrated system for water quality monitoring and management in wastewater treatment plants. Water.

[43] Jayaraman P., Nagarajan K.K., Partheeban P., Krishnamurthy V. (2024). Critical review on water quality analysis using IoT and machine learning models. International Journal of Information Management Data Insights.

[44] Crepaldi P.C., Pimenta T.C. (2017). Radio Frequency Identification.

[45] Sriskanthan N., Tan F., Karande A. (2002). Bluetooth based home automation system. Microprocess. Microsyst..

[46] Keertikumar M., Shubham M., Banakar R. (2015). 2015 International Conference on Green Computing and Internet of Things (ICGCIoT).

[47] Choi H.S., Carpenter D., Ko M.S. (2021). Risk taking behaviors using public wi-fi. Inf. Syst. Front.

[48] Gawas A.U. (2015). An overview on evolution of mobile wireless communication networks: 1g-6g. International Journal on Recent and Innovation Trends in Computing and Communication.

[49] Malik P., Bilandi N., Gupta A. (2022).

[50] Ijaradar, J., Chatterjee, S., . Real-Time Water Quality Monitoring System vol. 5, 6.

[51] Jha B.K. (2020). Cloud-based smart water quality monitoring system using IoT sensors and machine learning. Int. J. Adv. Trends Comput. Sci. Eng..

[52] Srivastava S., Vaddadi S., Sadistap S. (2018). Smartphone-based System for water quality analysis. Appl. Water Sci..

[53] Mabrouki J., Azrour M., Farhaoui Y., El Hajjaji S., Farhaoui Y. (2020).

[54] Jindal H., Saxena S., Kasana S.S. (2017). Sewage water quality monitoring framework using multi-parametric sensors. Wireless Pers. Commun..

[55] Jo W., Hoashi Y., Paredes Aguilar L.L., Postigo-Malaga M., Garcia-Bravo J.M., Min B.C. (2019). A low-cost and small USV platform for water quality monitoring. HardwareX.

[56] Rekhis S., Ellouze N., Boudriga N. (2012). Proceedings of the 8h ACM Symposium on QoS and Security for Wireless and Mobile Networks - Q2SWinet ’12.

[57] Postolache O., Girão P.S., Pereira J.M.D., Mukhopadhyay S.C., Lay-Ekuakille A., Fuchs A. (2011).

[58] Cook B.S., Cooper J.R., Kim S., Tentzeris M.M. (2013). 2013 IEEE MTT-S International Microwave Symposium Digest (MTT).

[59] Kamaludin K.H., Ismail W. (2017).

[60] Cook B.S., Vyas R., Kim S., Thai T., Le T., Traille A., Aubert H., Tentzeris M.M. (2014). RFID-based sensors for zero-power autonomous wireless sensor networks. IEEE Sensor. J..

[61] Chen Y., Han D. (2018). Water quality monitoring in smart city: a pilot project. Autom. ConStruct..

[62] Parameswari M., Moses M.B. (2018). Online measurement of water quality and reporting system using prominent rule controller based on aqua care-IOT. Des. Autom. Embed. Syst..

[63] Melo M., Mota F., Albuquerque V., Alexandria A. (2019). Development of a robotic airboat for online water quality monitoring in lakes. Robotics.

[64] Usha Kumari C., Laxmi Lydia E., Sampath Dakshina Murthy A., Kumar M. (2020). Designing of wireless sensor nodes for providing good quality drinking water to the public. Mater. Today: Proc..

[65] Al-Dahoud A., Fezari M., Mehamdia H., Zamojski W., Mazurkiewicz J., Sugier J., Walkowiak T., Kacprzyk J. (2020).

[66] Ionel R., Pitulice L., Vasiu G., Mischie S., Bizerea Spiridon O. (2015). Implementation of a GPRS based remote water quality analysis instrumentation. Measurement.

[67] Adamo F., Attivissimo F., Guarnieri Calo Carducci C., Lanzolla A.M.L. (2015). A smart sensor network for sea water quality monitoring. IEEE Sensor. J..

[68] Saravanan K., Anusuya E., Kumar R., Son L.H. (2018). Real-time water quality monitoring using Internet of Things in SCADA. Environ. Monit. Assess..

[69] Esakki B., Ganesan S., Mathiyazhagan S., Ramasubramanian K., Gnanasekaran B., Son B., Park S.W., Choi J.S. (2018). Design of amphibious vehicle for unmanned mission in water quality monitoring using internet of things. Sensors.

[70] Huan J., Li H., Wu F., Cao W. (2020). Design of water quality monitoring system for aquaculture ponds based on NB-IoT. Aquacult. Eng..

[71] Madeo D., Pozzebon A., Mocenni C., Bertoni D. (2020). A low-cost unmanned surface vehicle for pervasive water quality monitoring. IEEE Trans. Instrum. Meas..

[72] Simitha K., Subodh Raj M. (2019). 2019 3rd International Conference on Electronics, Communication and Aerospace Technology (ICECA).

[73] Di Gennaro P., Lofú D., Vitanio D., Tedeschi P., Boccadoro P. (2019). WaterS: a Sigfox-compliant prototype for water monitoring. Internet Technology Letters.

[74] Boccadoro P., Daniele V., Di Gennaro P., Lofù D., Tedeschi P. (2022). Water quality prediction on a Sigfox-compliant IoT device: the road ahead of WaterS. Ad Hoc Netw..

[75] Silva S., Nguyen Hoang Nghia, Tiporlini V., Alameh K. (2011). 8th International Conference on High-Capacity Optical Networks and Emerging Technologies.

[76] Lee S.W., Sarp S., Jeon D.J., Kim J.H. (2015). Smart water grid : the future water management platform. Desalination Water Treat..

[77] Rahu, Ahmed Mushtaque (2024). An IoT and machine learning solutions for monitoring agricultural water quality: a robust framework. Mehran Univ. Res. J. Eng. Technol..

[78] González Luis (2024). A low-cost IoT architecture based on LPWAN and MQTT for monitoring water resources in andean wetlands. SN Computer Science.

[79] Jamroen C., Yonsiri N., Odthon T., Wisitthiwong N., Janreung S. (2023). A standalone photovoltaic/battery energy-powered water quality monitoring system based on narrowband internet of things for aquaculture: design and implementation. Smart Agricultural Technology.

[80] Mansor Z., Abdul Latiff N.N.S. (2023). Materials Innovations and Solutions in Science and Technology: with a Focus on Tropical Plant Biomaterials.

[81] Gupta A.D., Islam M.M., Islam M.R., Sadek Z., Toha T.R., Mondol A., Alam S.M.M. (2023). 2023 International Conference on Electrical, Computer and Communication Engineering (ECCE).

[82] Razman N., Wan Ismail W., Abd Razak M., Ismail I., Jamaludin J. (2023). Design and analysis of water quality monitoring and filtration system for different types of water in Malaysia. Int. J. Environ. Sci. Technol..

[83] Rao A.P., Sarman K.G., Kumar G.V.P., Yerra S.D. (2023). Cognitive Computing and Cyber Physical Systems: Third EAI International Conference, IC4S 2022, Virtual Event, November 26-27, 2022, Proceedings.

[84] Murugan T.M., Shankar R.K., Shivkumar P., Kumar S.R., Gayathri K., Jeyam A. (2023). Monitoring and controlling the desalination plant using iot. Measurement: Sensors.

[85] Li Y., Wang X., Zhao Z., Han S., Liu Z. (2020). Lagoon water quality monitoring based on digital image analysis and machine learning estimators. Water Res..

[86] Akhter F., Siddiquei H.R., Alahi M.E.E., Jayasundera K.P., Mukhopadhyay S.C. (2022). An IoT-enabled portable water quality monitoring system with MWCNT/PDMS multifunctional sensor for agricultural applications. IEEE Internet Things J..

[87] Hartono R., Safi’Ie M.A., Yoeseph N.M., Bawono S.A.T., Hartatik Aziz A. (2022). 2022 1st International Conference on Smart Technology.

[88] Savel V., Rakluea P., Jangjing T., Kumkhet B., Mahatthanajatuphat C., Thaiwirot W. (2022). 2022 International Electrical Engineering Congress.

[89] Sendra S., Parra L., Jimenez J.M., Garcia L., Lloret J. (2022).

[90] Fonseca-Campos J., Reyes-Ramirez I., Guzman-Vargas L., Fonseca-Ruiz L., Mendoza-Perez J.A., Rodriguez-Espinosa P.F. (2022). Multipara- metric system for measuring physicochemical variables associated to water quality based on the Arduino platform. IEEE Access.

[91] Abidin Z., Maulana E., Nurrohman M.Y., Candra Wardana F., Warsito (2022). 2022 International Electronics Symposium (IES).

[92] Zeng J., Luo X.G., Wang Y., Liu Y., Zhang Z. (2022). 2022 5th International Conference on Pattern Recognition and Artificial Intelligence (PRAI).

[93] Baghel L.K., Gautam S., Malav V.K., Kumar S. (2022). TEMPSENSE: LoRa enabled integrated sensing and localization solution for water quality monitoring. IEEE Trans. Instrum. Meas..

[94] Wang S., Peng H., Liang S. (2022). Prediction of estuarine water quality using interpretable machine learning approach. J. Hydrol..

[95] Lloret J., García L., Jimenez J.M., Sendra S., Lorenz P. (2021). Cluster-based communication protocol and architecture for a wastewater purification system intended for irrigation. IEEE Access.

[96] Mubarak Y., Nyitamen D., Na’inna A. (2021). 2021 1st International Conference on Multidisciplinary Engineering and Applied Science.

[97] Tahatahir S.N.S., Talip M.S.A., Mohamad M., Hasan Z.H.A., Mohamad Z.F., Merican A.F.M.A., Othman M., Izam T.F.T.M.N., Salleh M.F.M. (2021). 2021 International Conference on Smart City and Green Energy.

[98] Liloja, Sreelekha M., Gopakumar G., Shahil K., Smys S., Palanisamy R., Rocha, Beligiannis G.N. (2021). Computer Networks and Inventive Communication Technologies.

[99] Nurwarsito H., Christian R.D. (2021). 2021 2nd International Conference on ICT for Rural Development.

[100] Raditya M., Darwito P.A., Ansori A.S., Kalahari B., Saputro P.G., Mufidah H. (2021). 2021 3rd International Conference on Research and.

[101] Lee C.W., Jeong H., Ryu J.H., Park J., Choi B.C. (2021). 2021 International Conference on Information and Communication Technology Convergence.

[102] Philip M.S., Singh P. (2021). Adaptive transmit power control algorithm for dynamic LoRa nodes in water quality monitoring system. Sustainable Computing: Informatics and Systems.

[103] Haque, H., Labeeb, K., Riha, R.B., Khan, M.N.R., 2021. IoT Based Water Quality Monitoring System By Using Zigbee Protocol, in:2021 International Conference on Emerging Smart Computing and Informatics (ESCI), IEEE, Pune, India. pp. 619–622. URL: https://ieeexplore.ieee.org/document/9397031/, doi:10.1109/ESCI50559.2021.9397031.

[104] Hsieh C.W., Tsai Y.J., Stefanie C., Wang C.C., Chang W.T.S. (2020). 2020 International Symposium on Computer, Consumer and Control.

[105] Alset U., Kulkarni A., Mehta H. (2020). Performance Analysis of Various LoRaWAN Frequencies For Optimal Data Transmission Of Water Quality Parameter Measurement, in: 2020 11th International Conference on Computing, Communication and Networking Technologies (ICCCNT), IEEE, Kharagpur.

[106] Ann Roseela J., Godhavari T., Narayanan R., Madhuri P. (2021). Design and deployment of IoT based underwater wireless communication system using electronic sensors and materials. Mater. Today: Proc..

[107] Sarnin S.S., Hussein A.B., Zahidi D.B., Naim N.F., Abdul Kadir R.S.B.S., Tan M.N.M. (2020). IEEE 5th International Symposium on Telecommunication Technologies (ISTT).

[108] Arvind A., Paul R., Bhulania P. (2020). 2020 7th International Conference on Signal Processing and Integrated Networks (SPIN).

[109] Bai Q., Wu J., Jin C. (2020). 2020 13th International Symposium on Computational Intelligence and Design (ISCID).

[110] Yunfeng L., Tianpei Z. (2019). 2019 International Conference on Smart Grid and Electrical Automation.

[111] Chowdury M.S.U., Emran T.B., Ghosh S., Pathak A., Alam M.M., Absar N., Andersson K., Hossain M.S. (2019). IoT based real-time river water quality monitoring system. Proc. Comput. Sci..

[112] Aggarwal S., Gulati R., Tech B., Bhushan B. (2019).

[113] Budiarti R.P.N., Sukaridhoto S., Hariadi M., Purnomo M.H. (2019). 2019 International Conference on Computer Science, Information Technology, and Electrical Engineering (ICOMITEE).

[114] Wu N., Khan M. (2019). 2019 SoutheastCon.

[115] Ngom B., Diallo M., Gueye B., Marilleau N. (2019). LoRa-based Measurement Station for Water Quality Monitoring: Case of Botanical Garden Pool.

[116] Manoharan A.M., Rathinasabapathy V. (2018). 2018 2nd International Conference on Smart Grid and Smart Cities.

[117] Liu Y.T., Lin B.Y., Yue X.F., Cai Z.X., Yang Z.X., Liu W.H., Huang S.Y., Lu J.L., Peng J.W., Chen J.Y. (2018). A solar powered long range real-time water quality monitoring system by LoRaWAN, in: 2018 27th Wireless and Optical Communication Conference (WOCC), IEEE, Hualien.

[118] Suryawanshi V., Khandekar M. (2018). 2018 Second International Conference on Intelligent Computing and Control Systems (ICICCS).

[119] Priya S.K., Shenbagalakshmi G., Revathi T. (2018). 2018 International Conference on Communication and Signal Processing (ICCSP).

[120] Puneeth K.M., Bipin S., Prasad C., Jathin Kumar R., Urs M.K. (2018). 2018 3rd IEEE International Conference on Recent Trends in Electronics, Information & Communication Technology (RTEICT).

[121] Das B., Jain P. (2017). 2017 International Conference on Computer, Communications and Electronics.

[122] Khaleeq H., Abou-ElNour A., Tarique M. (2016).

[123] Kafli N., Isa K. (2017). IEEE 7th International Conference on Underwater System Technology: Theory and Applications (USYS), IEEE, Kuala Lumpur.

[124] Salim T.I., Alam H.S., Pratama R.P., Anto I.A.F., Munandar A. (2017). 2017 2nd International Conference on Automation, Cognitive Science, Optics, Micro Electro-Mechanical System, and Information Technology (ICACOMIT).

[125] Myint C.Z., Gopal L., Aung Y.L. (2017). IEEE/ACIS 16th International Conference on Computer and Information Science (ICIS).

[126] Cloete N.A., Malekian R., Nair L. (2016). Design of smart sensors for real-time water quality monitoring. IEEE Access.

[127] Raut V., Shelke S. (2016). 2016 Conference on Advances in Signal Processing (CASP).

[128] Wiranto G., Maulana Y.Y., Hermida I.D.P., Syamsu I., Mahmudin D. (2015). International Conference on Smart Sensors and Application (ICSSA), IEEE, Kuala Lumpur, Malaysia.

[129] Mitchell T.M. (2006).

[130] Hastie T., Tibshirani R., Friedman J. (2009). The Elements of Statistical Learning.

[131] Quinlan J.R. (1986). Induction of decision trees. Mach. Learn..

[132] Nhaila H., Elmaizi A., Sarhrouni E., Hammouch A. (2019). Information Systems and Technologies to Support Learning: Proceedings of EMENA-ISTL 2018 2.

[133] Tian Y., Pan G., Alouini M.-S. (2021). Applying deep-learning-based computer vision to wireless communications : methodologies, opportunities, and challenges. IEEE Open Journal of the Communications Society.

[134] Nhaila H., Elmaizi A., Sarhrouni E., Hammouch A. (2019). Supervised classification methods applied to airborne hyperspectral images: comparative study using mutual information. Proc. Comput. Sci..

[135] Nhaila H., Sarhrouni E., Hammouch A. (2018). A new filter for dimensionality reduction and classification of hyperspectral images using GLCM features and mutual information. Int. J. Signal Imag. Syst. Eng..

[136] Alanazi A.H., Cradock A., Ryan J., Rainford L. (2022). Machine learning and deep learning-based natural language processing for auto-vetting the appropriateness of lumbar spine magnetic resonance imaging referrals. Inform. Med. Unlocked.

[137] Zourhri M., Hamida S., Akouz N., Cherradi B., Nhaila H., El Khaili M. (2023, May). 2023 3rd International Conference on Innovative Research in Applied Science, Engineering and Technology (IRASET).

[138] Pham Q.B., Mohammadpour R., Linh N.T.T., Mohajane M., Pourjasem A., Sammen S.S., Anh D.T., Nam V.T. (2021). Application of soft computing to predict water quality in wetland. Environ. Sci. Pollut. Control Ser..

[139] Abba S.I., Pham Q.B., Saini G., Linh N.T.T., Ahmed A.N., Mohajane M., Khaledian M., Abdulkadir R.A., Bach Q.V. (2020). Implementation of data intelligence models coupled with ensemble machine learning for prediction of water quality index. Environ. Sci. Pollut. Control Ser..

[140] Kim C.M., Parnichkun M. (2017). Prediction of settled water turbidity and optimal coagulant dosage in drinking water treatment plant using a hybrid model of k-means clustering and adaptive neuro-fuzzy inference system. Appl. Water Sci..

[141] Imani M., Hasan M.M., Bittencourt L.F., McClymont K., Kapelan Z. (2021).

[142] Deng T., Chau K.W., Duan H.F. (2021). Machine learning based marine water quality prediction for coastal hydro-environment management. J. Environ. Manag..

[143] Liu M., Lu J. (2014). Support vector machine—an alternative to artificial neuron network for water quality forecasting in an agricultural nonpoint source polluted river?. Environ. Sci. Pollut. Control Ser..

[144] Guo H., Huang J.J., Chen B., Guo X., Singh V.P. (2021). A machine learning-based strategy for estimating non-optically active water quality parameters using Sentinel-2 imagery. Int. J. Rem. Sens..

[145] El Bilali A., Taleb A., Brouziyne Y. (2021). Groundwater quality forecasting using machine learning algorithms for irrigation purposes. Agric. Water Manag..

[146] Lu H., Ma X. (2020). Hybrid decision tree-based machine learning models for short-term water quality prediction. Chemosphere.

[147] Heddam S., Kisi O. (2017). Extreme learning machines: a new approach for modeling dissolved oxygen (DO) concentration with and without water quality variables as predictors. Environ. Sci. Pollut. Control Ser..

[148] Chen H., Chen A., Xu L., Xie H., Qiao H., Lin Q., Cai K. (2020). A deep learning CNN architecture applied in smart near-infrared analysis of water pollution for agricultural irrigation resources. Agric. Water Manag..

[149] Ewusi A., Ahenkorah I., Aikins D. (2021). Modelling of total dissolved solids in water supply systems using regression and supervised machine learning approaches. Appl. Water Sci..

[150] Barzegar R., Asghari Moghaddam A., Adamowski J., Ozga-Zielinski B. (2018). Multi-step water quality forecasting using a boosting ensemblemulti-wavelet extreme learning machine model. Stoch. Environ. Res. Risk Assess..

[151] Wang L., Zhu Z., Sassoubre L., Yu G., Liao C., Hu Q., Wang Y. (2021). Improving the robustness of beach water quality modeling using an ensemble machine learning approach. Sci. Total Environ..

[152] Li Y., Wang X., Zhao Z., Han S., Liu Z. (2020). Lagoon water quality monitoring based on digital image analysis and machine learning estimators. Water Res..

[153] Hinton G.E., Sejnowski T.J. (1999). Unsupervised Learning: Foundations of Neural Computation.

[154] Sundar, Balakrishnan P.V., Cooper M.C., Jacob V.S., Lewis P.A. (1994). A study of the classification capabilities of neural networks using unsupervised learning: a comparison withK-means clustering. Psychometrika.

[155] Wang Y., Wai K.P., Chia M.Y., Koo C.H., Huang Y.F., Chong W.C. (2022). Applications of deep learning in water quality management : a state-of-the-art review. J. Hydrol..

[156] Krishnaraj A., Deka P.C. (2020). Spatial and temporal variations in river water quality of the Middle Ganga Basin using unsupervised machine learning techniques. Environ. Monit. Assess..

[157] Sun A.Y., Scanlon B.R. (2019). How can Big Data and machine learning benefit environment and water management : a survey of methods, applications, and future directions. Environ. Res. Lett..

[158] Cao X., Liu Y., Wang J., Liu C., Duan Q. (2020). Prediction of dissolved oxygen in pond culture water based on K-means clustering and gated recurrent unit neural network. Aquacult. Eng..

[159] Haghnazar H., Johannesson K.H., González-Pinzón R., Pourakbar M., Aghayani E., Rajabi A., Hashemi A.A. (2022). Groundwater geochemistry, quality, and pollution of the largest lake basin in the Middle East: comparison of PMF and PCA-MLR receptor models and application of the source-oriented HHRA approach. Chemosphere.

[160] Al-Sulttani A.O., Al-Mukhtar M., Roomi A.B., Farooque A.A., Khedher K.M., Yaseen Z.M. (2021). Proposition of new ensemble data- intelligence models for surface water quality prediction. IEEE Access.

[161] Visalakshi S., Radha V., Jain L.C., Behera H.S., Mandal J.K., Mohapatra D.P. (2015). Computational Intelligence in Data Mining - Volume 1.

[162] Mohammadrezapour O., Kisi O., Pourahmad F. (2020). Fuzzy c-means and K-means clustering with genetic algorithm for identification of homogeneous regions of groundwater quality. Neural Comput. Appl..

[163] Meng L., Zuo R., Wang J.s., Yang J., Teng Y.g., Shi R.t., Zhai Y.z. (2018). Apportionment and evolution of pollution sources in a typical riverside groundwater resource area using PCA-APCS-MLR model. J. Contam. Hydrol..

[164] Zhang H., Cheng S., Li H., Fu K., Xu Y. (2020). Groundwater pollution source identification and apportionment using PMF and PCA- APCA-MLR receptor models in a typical mixed land-use area in Southwestern China. Sci. Total Environ..

[165] Jamshidzadeh Z., Latif S.D., Ehteram M., Sheikh Khozani Z., Ahmed A.N., Sherif M., El-Shafie A. (2024). An advanced hybrid deep learning model for predicting total dissolved solids and electrical conductivity (EC) in coastal aquifers. Environ. Sci. Eur..

[166] Uddin M.G., Nash S., Rahman A., Olbert A.I. (2023). A novel approach for estimating and predicting uncertainty in water quality index model using machine learning approaches. Water Res..

[167] Hu G., Mian H.R., Mohammadiun S., Rodriguez M.J., Hewage K., Sadiq R. (2023). Appraisal of machine learning techniques for predicting emerging disinfection byproducts in small water distribution networks. J. Hazard Mater..

[168] Omeka M.E. (2023). Environmental Science and Pollution Research URL.

[169] Uddin M.G., Nash S., Rahman A., Olbert A.I. (2023). Performance analysis of the water quality index model for predicting water state using machine learning techniques. Process Saf. Environ. Protect..

[170] Lap B.Q., Phan T.T.H., Nguyen H.D., Quang L.X., Hang P.T., Phi N.Q., Hoang V.T., Linh P.G., Hang B.T.T. (2023). Predicting Water QualityIndex (WQI) by feature selection and machine learning: a case study of an Kim Hai irrigation system. Ecol. Inf..

[171] Yan T., Zhou A., Shen S.L. (2023). Prediction of long-term water quality using machine learning enhanced by Bayesian optimisation. Environ. Pollut..

[172] Narita K., Matsui Y., Matsushita T., Shirasaki N. (2023). Screening priority pesticides for drinking water quality regulation and monitoring by machine learning: analysis of factors affecting detectability. J. Environ. Manag..

[173] Chen P., Wang B., Wu Y., Wang Q., Huang Z., Wang C. (2023). Urban river water quality monitoring based on self-optimizing machine learning method using multi-source remote sensing data. Ecol. Indicat..

[174] Nasir N., Kansal A., Alshaltone O., Barneih F., Sameer M., Shanableh A., Al-Shamma’a A. (2022). Water quality classification using machine learning algorithms. J. Water Proc. Eng..

[175] Azrour M., Mabrouki J., Fattah G., Guezzaz A., Aziz F. (2022). Machine learning algorithms for efficient water quality prediction. Modeling Earth Systems and Environment.

[176] Uddin M.G., Nash S., Mahammad Diganta M.T., Rahman A., Olbert A.I. (2022). Robust machine learning algorithms for predicting coastal water quality index. J. Environ. Manag..

[177] Khullar S., Singh N. (2022). Water quality assessment of a river using deep learning Bi-LSTM methodology: forecasting and validation. Environ. Sci. Pollut. Control Ser..

[178] Nourani V., Ghaneei P., Kantoush S.A. (2022). Robust clustering for assessing the spatiotemporal variability of groundwater quantity and quality. J. Hydrol..

[179] Zai C., El Mechal C., El Amrani El Idrissi N., Ghennioui H., Motahhir S., Bossoufi B. (2022). Digital Technologies and Applications.

[180] Kadkhodazadeh M., Farzin S. (2022). Introducing a novel hybrid machine learning model and developing its performance in estimating water quality parameters. Water Resour. Manag..

[181] Kokatnoor S.A., Reddy V., Balachandran K., Saraswat M., Sharma H., Balachandran K., Kim J.H., Bansal J.C. (2022). Congress on Intelligent Systems.

[182] Gómez D., Salvador P., Sanz J., Casanova J.L. (2021). A new approach to monitor water quality in the Menor sea (Spain) using satellite data and machine learning methods. Environ. Pollut..

[183] Tousi E.G., Duan J.G., Gundy P.M., Bright K.R., Gerba C.P. (2021). Evaluation of E. coli in sediment for assessing irrigation water quality using machine learning. Sci. Total Environ..

[184] Kouadri S., Elbeltagi A., Islam A.R.M.T., Kateb S. (2021). Performance of machine learning methods in predicting water quality index based on irregular data set: application on Illizi region (Algerian southeast). Appl. Water Sci..

[185] Xu T., Coco G., Neale M. (2020). A predictive model of recreational water quality based on adaptive synthetic sampling algorithms and machine learning. Water Res..

[186] Bui D.T., Khosravi K., Tiefenbacher J., Nguyen H., Kazakis N. (2020). Science of the Total Environment 721.

[187] Radhakrishnan N., Pillai A.S. (2020). 2020 5th International Conference on Communication and Electronics Systems (ICCES).

[188] Jalal D., Ezzedine T. (2020). 2020 International Wireless Communications and Mobile Computing (IWCMC).

[189] Muharemi F., Logofătu D., Leon F. (2019). Machine learning approaches for anomaly detection of water quality on a real-world data set. Journal of Information and Telecommunication.

[190] Najah Ahmed A., Binti Othman F., Abdulmohsin Afan H., Khaleel Ibrahim R., Ming Fai C., Shabbir Hossain M., Ehteram M., Elshafie A. (2019). Machine learning methods for better water quality prediction. J. Hydrol..

[191] Cao S., Wang S., Zhang Y. (2018). 2018 17th IEEE International Conference on Machine Learning and Applications (ICMLA).

[192] Chen Q., Cheng G., Fang Y., Liu Y., Zhang Z., Gao Y., Horn B.K.P. (2018). 2018 4th International Conference on Universal Village (UV).

[193] Dezfooli D., Hosseini-Moghari S.M., Ebrahimi K., Araghinejad S. (2018). Classification of water quality status based on minimum quality parameters: application of machine learning techniques. Modeling Earth Systems and Environment.

[194] Sharif S.M., Kusin F.M., Asha’ari Z.H., Aris A.Z. (2015). Characterization of water quality conditions in the klang River Basin, Malaysia using self organizing map and K-means algorithm. Procedia Environmental Sciences.

[195] Liu Fu-cheng, He Xue-zhao (2013). 2013 Fifth International Conference on Measuring Technology and Mechatronics Automation.

[196] Zhang Y., Love D.J., Krogmeier J.V., Anderson C.R., Heath R.W., Buckmaster D.R. (2021). Challenges and opportunities of future rural wireless communications. IEEE Commun. Mag..

[197] Wang B. (2011). Coverage problems in sensor networks : a survey. ACM Comput. Surv..

[198] Tshiluna N.B., Mathevula H.L., Rimer S., Pinifolo J., Paul B.S., Jayram S., Mikeka C. (2016).

[199] Jahankhani H., Kendzierskyj S., Akhgar B., Éds (2021).

[200] Aslam M.M., Tufail A., Kim K.-H., Apong R.A.A.H.M., Raza M.T. (2023). A comprehensive study on cyber attacks in communication networks in water purification and distribution plants : challenges, vulnerabilities, and future prospects. Sensors.

[201] Stellios I., Kotzanikolaou P., Psarakis M., Alcaraz C., Lopez J. (2018). A survey of IoT-enabled cyberattacks : assessing attack paths to critical infrastructures and services. IEEE Communications Surveys & Tutorials.

[202] Mounce S.R., Scozzari A., Mounce S., Han D., Soldovieri F., Solomatine D. (2020).

[203] Zhang Y., Liu W., Li Z., Li Z., Cheng H., Chen S., Tian J. (2018). High-quality-factor multiple Fano resonances for refractive index sensing. Opt Lett..

[204] Gupta M., Srivastava Y.K., Manjappa M., Singh R. (2017). Sensing with toroidal metamaterial. Appl. Phys. Lett..

[205] Padyab A., Habibipour A., Rizk A., Ståhlbröst A. (2019). Adoption barriers of IoT in large scale pilots. Information.

